# RecBCD coordinates repair of two ends at a DNA double-strand break, preventing aberrant chromosome amplification

**DOI:** 10.1093/nar/gky463

**Published:** 2018-06-13

**Authors:** Martin A White, Benura Azeroglu, Manuel A Lopez-Vernaza, A M Mahedi Hasan, David R F Leach

**Affiliations:** Institute of Cell Biology, School of Biological Sciences, University of Edinburgh, King's Buildings, Mayfield Road, Edinburgh EH9 3FF, UK

## Abstract

DNA double-strand break (DSB) repair is critical for cell survival. A diverse range of organisms from bacteria to humans rely on homologous recombination for accurate DSB repair. This requires both coordinate action of the two ends of a DSB and stringent control of the resultant DNA replication to prevent unwarranted DNA amplification and aneuploidy. In *Escherichia coli*, RecBCD enzyme is responsible for the initial steps of homologous recombination. Previous work has revealed *recD* mutants to be nuclease defective but recombination proficient. Despite this proficiency, we show here that a *recD* null mutant is defective for the repair of a two-ended DSB and that this defect is associated with unregulated chromosome amplification and defective chromosome segregation. Our results demonstrate that RecBCD plays an important role in avoiding this amplification by coordinating the two recombining ends in a manner that prevents divergent replication forks progressing away from the DSB site.

## INTRODUCTION

Organisms as distantly related as humans and bacteria have evolved sophisticated mechanisms to ensure the stable inheritance of their genomes through successive generations. However, genome instability does occur and takes the form of mutations, chromosome rearrangements, and both gene and whole chromosome number variations (aneuploidy). Genome instability is an enabling characteristic of cancer (1) and a direct cause of several human syndromes (e.g. Down syndrome and Klinefelter syndrome). These have been attributed to aberrant DNA replication (e.g. (2)), homologous recombination (e.g. (3)) and chromosome segregation (e.g. (4)). Importantly, genome instability *per se* need not be detrimental as aneuploidy is found in human hepatocytes (5), aneuploidy and copy number variations are found in neurons (6), and mosaic aneuploidy contributes to adaptability in organisms such as *Leishmania* (7). Since recombination involves DNA synthesis and can lead to chromosomal amplification and duplication via recombination-dependent replication in prokaryotes and break-induced replication in eukaryotes (8–11), it is interesting to consider how segregation errors resulting in aneuploidy and copy number variation may arise from improper interactions between recombination and DNA replication.

The recombination and DNA replication systems of *Escherichia coli* can provide insight into how chromosomes are handled correctly to avoid segregation errors and how such errors can arise when chromosomes are handled incorrectly. *Escherichia coli* DNA replication is initiated at a unique origin (*oriC*) and proceeds bi-directionally around the circular chromosome until the two replication forks meet in the terminus region (12). The two replicated copies of the chromosome are then segregated accurately to the daughter cells. We have developed a system where we can induce a replication-dependent DNA double-strand break (DSB) in the *lacZ* gene approximately half way around the chromosome on the right replichore (13). The DSB is generated by SbcCD-mediated cleavage of a DNA hairpin formed by a long palindrome on one of the sister chromosomes during replication and is accurately repaired by homologous recombination using the un-cleaved sister chromosome as a template. The palindrome-induced DSB repair (DSBR) reaction is remarkably efficient, causing a fitness loss of only 0.6% per generation in fast-growing cells (14). DNA repair is absolutely dependent on the action of RecBCD and RecA mediated homologous recombination causing *recB* and *recA* mutants to die when subjected to palindrome-induced DSBs (13).

The heterotrimeric RecBCD complex is a highly rapid and processive DNA helicase-nuclease that generates ssDNA at broken DNA ends upon which it actively loads RecA (15), the enzyme that catalyses DNA sequence homology-dependent strand exchange. RecB and RecD are helicases with opposite polarity (RecB, 3′ to 5′; RecD, 5′ to 3′). In addition to its helicase activity, RecB also possesses nuclease activity. This activity requires an interaction with RecD and is modulated upon binding of RecC to a polar octomeric sequence (5′-GCTGGTGG) known as Chi (16,17). RecBC can form a complex in the absence of RecD *in vitro*. In comparison to RecBCD, RecBC has reduced processivity (18), lacks exonuclease activity (19) and constitutively loads RecA independently of Chi (20).

In this work, we have analysed the behaviour of a *recD* null mutant that is expected to repair DSBs using RecBC enzyme. Previous studies have revealed that *recD* mutants are recombination proficient and even have a hyper-recombination (hyper-rec) phenotype in some assays (21) despite being nuclease defective (22). However, here we show that *recD* mutants are repair deficient when subjected to two-ended palindrome induced DSBs. Furthermore, we show that cells lacking RecD are unable to coordinate the two ends of the DSB and this results in aberrant DNA replication and defective chromosome segregation.

## MATERIALS AND METHODS

A detailed description of materials and experimental methodology can be found in supplementary file 1. A brief summary is provided below.

### Bacterial strains and growth conditions

**Figure 1.**
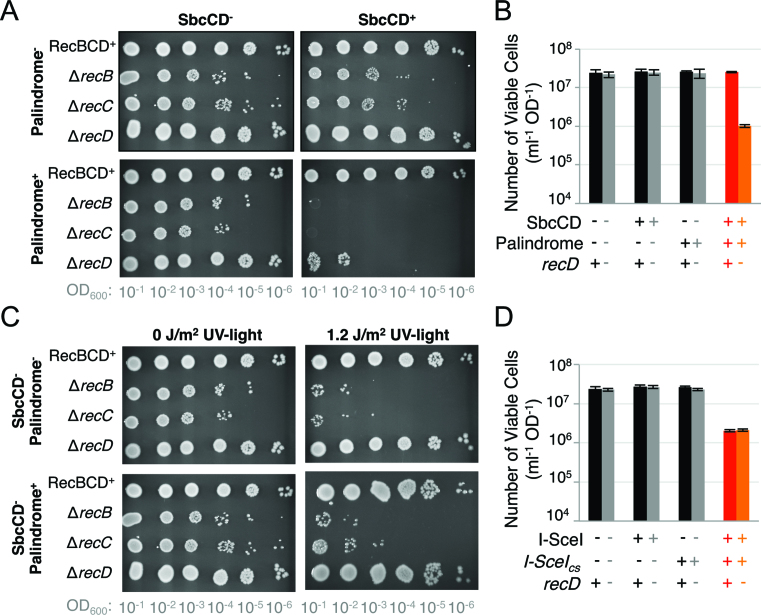
RecD is required for cells to survive certain forms of DNA damage. (**A**) Cultures of strains containing the DNA palindrome or not were serially diluted and grown overnight under either conditions where SbcCD was induced (SbcCD^+^), or repressed (SbcCD^−^). Colony formation at higher dilution (lower OD_600_) indicates increased cellular viability. (**B, D**) Quantitative assessment of the number of viable cells (the number of colony forming units per 1 ml of culture normalised to an optical density at 600 nm of 1) following 60 min of exponential growth with (B) SbcCD either expressed or not; (D) I-SceI either expressed or not. (**C**) Cultures of strains containing the DNA palindrome or not were serially diluted and grown overnight under either conditions where SbcCD was repressed following, or not, exposure to ultraviolet (UV)-light. Data for A) and C) are representative experiments; data for B) and D) are represented as mean ± S.E.M., *n* = 3. No difference in viability was detected between the *recD*^+^ I-SceI^+^*I-SceI_cs_^+^* and *recD*^−^ I-SceI^+^*I-SceI_cs_^+^* samples (two-sample *t* test, *P* = 0.68). See also Supplementary Figure S1.

All bacteria used in this study were derived from the non-pathogenic *E. coli* K12 strain BW27784. Expression of SbcCD and I-SceI was repressed or induced by the addition of glucose or arabinose at the indicated concentration. P*_sfiA_-gfp* expression and total cellular DNA content were assayed by flow cytometry following 2 h of growth in the presence of arabinose/glucose. For all other experiments (except growth curve, Supplementary Figure S1 and cellular viability Figure 1A), cells were sampled following 1 h of growth in the presence of arabinose/glucose. All materials used in this study are listed in Supplementary Table S1.

### PFGE and Southern blotting

Genomic DNA (gDNA) in agarose plugs was digested with I-SceI and fragments separated using a CHEF-DR II PFGE (Biorad) machine at 6 V/cm for 10 h in 0.5× TBE buffer at 4°C. DNA was then transferred to a positively charged nylon membrane by Southern blotting and cross-linked to the membrane using UV-light. Chromosome fragments of interest were detected using ^32^P α-dATP incorporated radiolabeled DNA probes. Probes were hybridized to membranes overnight at 65°C in 10 ml of Church-Gilbert buffer (7% SDS, 0.5 M NaH_2_PO_4_, 1 mM EDTA, 1% BSA), then washed twice at 60°C, first for 15 min in a solution of 0.3 M NaCl, 0.03 M Na citrate and 0.1% SDS, and then for 30 min in a solution of 0.075 M NaCl, 0.0075 M sodium citrate and 0.1% SDS.

### 2D gel electrophoresis and Southern blotting

gDNA was isolated as described above for PFGE (‘plug’ method), except cells were concentrated to an OD_600_ of 100 in TEN buffer prior to mixing with a 0.8% low melting point agarose in TEN buffer solution. Prior to restriction digest, gDNA (in plugs) was washed six times in appropriate restriction buffer for 1 h at room temperature with agitation, prior to overnight digestion at 37°C in restriction buffer with 600 units of restriction enzyme. Digested DNA was loaded onto a 0.4% agarose gel in 1× TBE (89 mM Tris-borate, 2 mM EDTA) at 1 V/cm for 24 h at 4°C. Lanes containing the separated DNA were cut out, rotated 90° and cast in a new gel composed of 1% agarose in 1× TBE supplemented with 0.3 μg ml^−1^ ethidium bromide. This gel (the second dimension) was ran at 6 V/cm for 15 h in circulating 1x TBE buffer supplemented with 0.3 μg ml^−1^ ethidium bromide at 4°C. DNA was then transferred to a positively charged nylon membrane by Southern blotting and cross-linked to the membrane using UV-light. Chromosome fragments of interest were detected using ^32^P α-dATP incorporated radiolabeled DNA probes as described for PFGE. Data were analysed as described in supplementary file 1.

### Chromosome marker frequency analysis

gDNA was isolated from 20 ml cultures using a Wizard Genomic DNA Purification Kit following manufacturer's guidelines. Purified gDNA was treated with the supplied RNase for 50 min and rehydrated overnight in the supplied TE buffer at 4°C. Three units of the RNase blend Riboshredder was then added to further destroy contaminating RNA. Samples were then purified again by phenol/chloroform extraction and ethanol precipitation. Libraries were prepared from the gDNA by Edinburgh Genomics using an Illumina TruSeq DNA Sample Prep kit. Edinburgh Genomics subsequently obtained paired-end reads of the samples using an Illumina HiSeq 2000 platform. Two biological repeats of each strain were acquired. Data were analysed as described in supplementary file 1.

### RecA ChIP-seq

Cells were fixed by the addition of formaldehyde (final concentration 1%) for 10 min at 22.5°C to crosslink proteins to DNA. Crosslinking was quenched by the addition of 0.5 M glycine. Cells were then collected by centrifugation at 1500 × g for 7 min before washing three times in ice-cold 1× PBS and re-suspending in 250 μl of ChIP buffer (10 ml ChIP buffer consists of 200 mM Tris–HCl (pH 8.0), 600 mM NaCl, 4% Triton X and 1 cOmplete™ protease inhibitor cocktail EDTA-free tablet). Samples were then sonicated using a Diagenode Bioruptor^®^ at 30 s intervals for 10 min at high amplitude. After sonication, 350 μl of ChIP buffer was added to each sample and the samples gently mixed by pipetting. Immunoprecipitation was performed overnight at 4°C using 1/100 anti-RecA antibody (Abcam, ab63797). Immunoprecipitated samples were then incubated with Protein G Dynabeads^®^ for 2 h with rotation at room temperature. All samples were washed three times with 1× PBS + 0.02% Tween-20 before re- suspending the Protein G Dynabeads^®^ in 200 μl of TE buffer + 1% SDS. 100 μl of TE buffer + 1% SDS were added to the input samples and all samples were then incubated at 65°C for 10 h to reverse the formaldehyde cross-links. DNA was isolated using the MinElute PCR purification kit according to manufacturer's instructions. DNA was eluted in 100 μl of TE buffer using a two-step elution. Samples were stored at –20°C. Two biological repeats of each strain were acquired. Libraries of the immunoprecipitated DNA were made using NEBNext^®^ ChIP-Seq library preparation kit. Briefly, the samples were first subjected to end repair to fill in ssDNA overhangs, remove 3′ phosphates and phosphorylate the 5′ ends of DNA. Klenow exo- was used to adenylate the 3′ ends of the DNA and NEBNext DNA adaptors (provided in the NEBNext Multiplex Oligos for Illumina kit) were ligated using T4 DNA ligase. After each step, the DNA was purified using the Qiagen MinElute PCR purification kit according to the manufacturer's instructions. After adaptor ligation, the adaptor-modified DNA fragments were enriched by PCR using primers (provided in the NEBNext Multiplex Oligos for Illumina kit) corresponding to the beginning of each adaptor. Finally, agarose gel electrophoresis was used to size select adaptor-ligated DNA with an average size of approximately 300 bp. All samples were quantified on a Bioanalyzer (Agilent) before being sequenced on either an Illumina^®^ HiSeq 2500 (for DL4184, DL4201 and DL5699) or HiSeq 4000 (for DL6204) by Edinburgh Genomics. Data were analysed as described in supplementary file 1.

### Flow cytometry

To determine SOS induction from *P_sfiA_-gfp*, GFP fluorescence was measured using an Apogee A50 flow cytometer. Either two or three biological repeats of each strain and condition were acquired. Relative cellular DNA contents were measured in fixed cells by adding 1 ml of culture to 8 ml of 100% ethanol. Fixed cells were collected by centrifugation, washed twice in 1× PBS and re-suspended in 400 μl of 1× PBS. RNA was then degraded and DNA stained by the addition of 100 μl of a 1× PBS, 50 μg ml^−1^ propidium iodide, 500 μl ml^−1^ RNaseA solution. Propidium iodide fluorescence (a measure of DNA content) was measured using an Apogee A50 flow cytometer. Three biological repeats of each strain and condition were acquired.

### Microscopy

Cells were grown in M9 minimal growth media supplemented with 0.2% glycerol and 1 ng ml^−1^ anhydrotetracycline at 37°C. 10 μl of cell culture was mounted on a pad of 1% agarose.H_2_O, covered with #1.5 coverslip and imaged by widefield fluorescence microscopy at a resolution of 100 nm X, 100 nm Y, 200 nm Z using a Zeiss Axiovert 200 fluorescence microscope equipped with a 100× Objective NA1.4 phase objective with a 1.6× Optivar, Photometrics Evolve 512 EMCCD camera, Xenon light source and piezo stage. The microscope was controlled using Metamorph software. Acquired images were deconvolved using Autoquant X2 and visualized and processed using FIJI. Three biological repeats were acquired for each strain.

## RESULTS

### All three subunits of the RecBCD complex are required to survive cleavage of a DNA hairpin by SbcCD nuclease

In contrast to *recB* and *recC* mutants that are known to be defective in homologous recombination (e.g. (13)), *recD* mutants have been characterised as recombination proficient (or even hyper-rec) in P1 transduction, Hfr mating and *red gam* bacteriophage lambda crosses (21). It was therefore surprising that all three subunits of the RecBCD complex were required for cells to survive in the presence of an SbcCD-induced DSB (Figure 1A). In contrast to cultures of RecBCD^+^ cells, 1 h of SbcCD expression in cultures of Δ*recD* mutants harbouring a 246 bp interrupted DNA palindrome in the *lacZ* gene of the *E. coli* chromosome resulted in a 96% decrease in the number of viable cells (Figure 1B); after 2 h of SbcCD expression cell mass ceased to increase (Supplementary Figure S1). Consistent with published data on *recD* mutants, these Δ*recD* cells (unlike the Δ*recB and* Δ*recC* cells) were able to survive DNA damage induced by ultra-violet light (Figure 1C), indicating that the observed DNA repair defect is not a result of the Δ*recD* allele used in this study. Site-specific DSBs induced at the same *lacZ* locus by the homing endonuclease I-SceI resulted in an approximate 10-fold decrease in viability of both RecBCD^+^ and Δ*recD* cells (Figure 1D). The Δ*recD* cells were not found to be more sensitive to an I-SceI induced DSB than RecBCD^+^ cells (*P* = 0.68, two-sample *t*-test).

### DSB repair in the absence of RecD results in asymmetric cell divisions, defective chromosome segregation, increased cellular DNA content and strong induction of the DNA damage response

**Figure 2.**
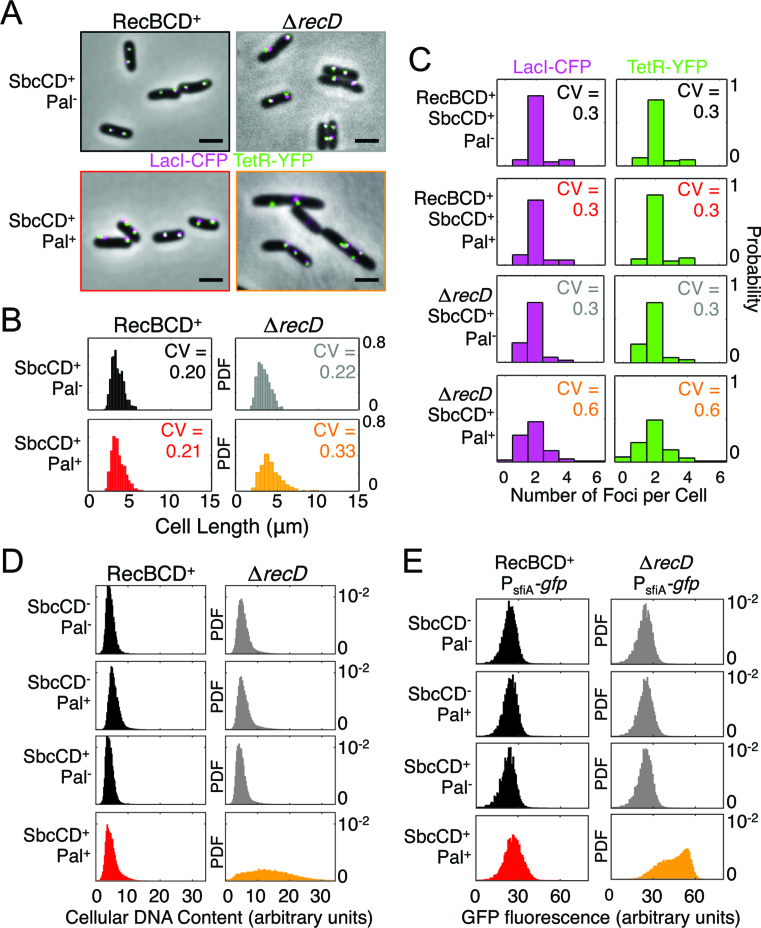
DSB repair in the absence of RecD results in altered cellular physiology. (**A**) Micrographs of RecBCD^+^ and Δ*recD* cells containing or not the DNA palindrome (Pal) following 60 min of SbcCD expression. Cells harboured an array of *tetO* and *lacO* sequences on either side of the palindrome integration site (*lacZ*) and expressed TetR-YFP (green) and LacI-CFP (magenta) from a constitutive promoter. Scale bar shows 5 μm. (**B**) Distribution of cell lengths and the coefficient of variation (CV). Data are for 3 biological repeats combined. (**C**) Histogram showing the distribution of number of detectable LacI-CFP and TetR-YFP foci per cell and the coefficient of variation (CV). Data are the cumulative data from 3 biological repeats. (**D**) Cellular DNA content of >100,000 cells was measured by flow cytometry following 2 hours of exponential growth with SbcCD expression either induced or repressed in cells containing the palindrome or not. Data is from a single representative experiment. (**E**) Expression of green fluorescence protein (GFP) from the DNA-damage inducible promoter P*_sfiA_* as measured by flow cytometry following two hours of exponential growth with SbcCD either expressed or not. At least 100 000 cells were measured for each sample. Data is from a single representative experiment. For (B–E), data are displayed as a probability distribution function (PDF) to normalize for differences in total number of analysed cells. See also Supplementary Figure S2.

To test the physiological consequence of the RecBC-dependent DSB repair, we assessed cellular morphology and chromosome organization by wide-field fluorescence imaging of strains harbouring an array of *tetO* and an array of *lacO* sequences on either side of the palindrome integration site *lacZ* (23) and expressing TetR-YFP and LacI-CFP from a constitutive promoter integrated in the chromosome as well as HupA-mCherry (a non-sequence specific DNA binding protein) expressed from the native *hupA* locus (Figure 2A). In contrast to RecBCD^+^ cells, the DSB resulted in a large increase in the variability of cell size (Figure 2B), the number of visibly segregated *lacZ* loci (Figure 2C) and altered nucleoid morphology (Supplementary Figure S2). Cells also lacked the regular repeating pattern of twin YFP/CFP foci observed in controls (Figure 2A and Supplementary Figure S2) and often contained invaginations (division septa) that were clearly displaced from mid-cell (e.g. Figure 2A). Although complex and variable in nature, these cellular phenotypes are all consistent with defective chromosome segregation.

DNA synthesis is an essential intermediate stage of DSBR. Total cellular DNA content was assayed by flow cytometry following fixation, RNase treatment and propidium iodide staining. Hairpin cleavage by SbcCD had no notable effect on cellular DNA content in RecBCD^+^ samples but resulted in a very broad distribution of DNA content in Δ*recD* cells, most of which possessed significantly more DNA than Δ*recD* cells that did not experience an induced DSB (Figure 2D).

A key intermediate in the DSB repair reaction, RecA protein bound to ssDNA, catalyses auto-cleavage of the transcriptional repressor LexA (24), resulting in the DNA damage response (known as the SOS response in *E. coli*). Induction of the SOS response can be detected using a reporter consisting of the green fluorescence protein gene placed under the SOS-inducible promoter P*_sfiA_* (25). To test if induction of a DSB induced the SOS response in the absence of RecD, we integrated a copy of this reporter into the *E. coli* chromosome and quantified cellular GFP fluorescence by flow cytometry. Consistent with a similar assay using a P*_sfiA_-gfp* reporter on a plasmid (14), cleavage of the hairpin by SbcCD in RecBCD^+^ cells resulted in a highly reproducible increase in GFP expression (Figure 2E). This DSB-dependent increase in cellular GFP accumulation was more dramatic, and possibly multi-modal, in the Δ*recD* background (Figure 2E).

### Broken DNA ends are recombinogenic in the absence of RecD

**Figure 3.**
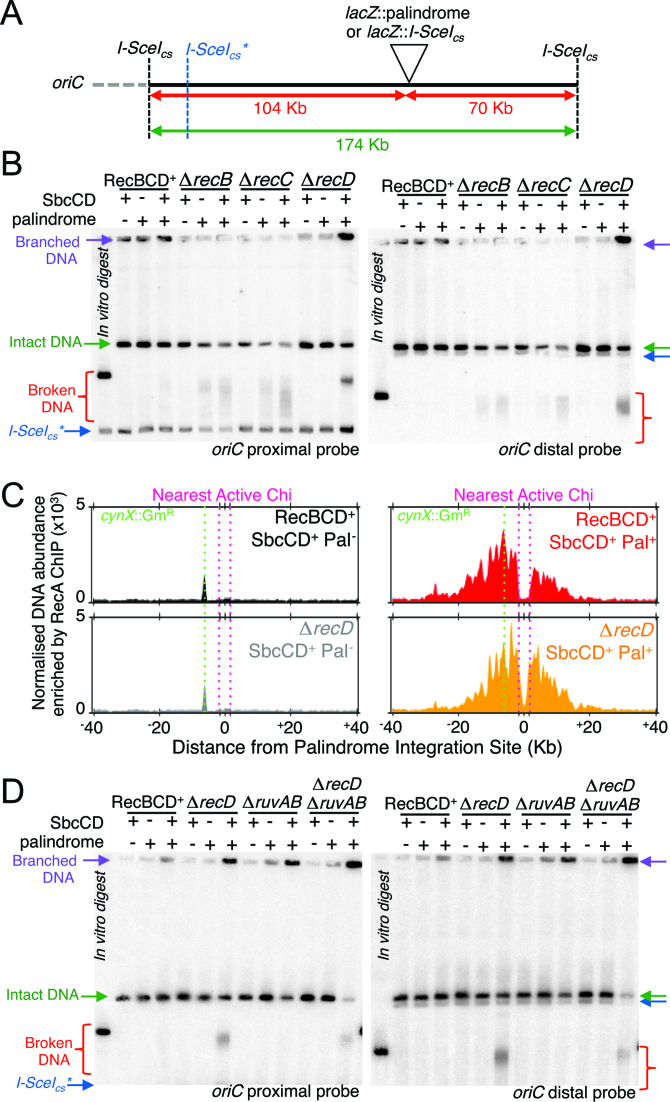
RecD is not required for recombination intermediate formation. (**A, B** and **D**) Analysis of the state of a 174 Kb DNA fragment containing the DSB locus (intact, broken or branched) by pulsed-field gel electrophoresis and Southern blot, probed for both sides of the DSB locus (proximal and distal to the origin of replication *oriC*) following induction of SbcCD expression for 60 min. DNA from a strain harbouring an I-SceI cleavage site in the locus in which the palindrome is integrated was digested with I-SceI *in vitro* to act as marker for broken DNA (‘*in vitro* digest’). *In vitro* digestion with I-SceI results in low frequency cleavage at an endogenous chromosomal site (I-SceI_cs_*). (**C**) Normalized enrichment of DNA in an 80 kb region containing the DSB locus following RecA chromatin immunoprecipitation (ChIP) in RecBCD+ and *recD* mutants with and without a palindrome at *lacZ*, following induction of SbcCD expression for 60 min. To account for variability in sequencing depth, data were normalized using a median of ratios scaling factor. The data was smoothed using a loess filter with a 2 kb window. All panels show results from single, representative experiments in which DNA replication proceeds from left to right. See also Supplementary Figure S3.

To test if a specific recombination intermediate accumulated in Δ*recD* cells subject to the DSB, we examined a 174 kb region flanking the DSB locus (*lacZ*) by pulsed-field gel electrophoresis (PFGE) and Southern blotting (Figure 3A). This large region of chromosomal DNA was selected due to the high processivity of RecBCD. In the presence of RecBCD, the DSB is repaired quickly and efficiently with no detectable alteration in DNA state at this level of resolution (Figure 3B, (13)). In contrast, in the absence of either RecB or RecC proteins, linear intact DNA was lost in response to the DSB and broken DNA accumulated as a range of sizes (Figure 3B, (13)). In the absence of RecD protein, cells subject to the DSB also accumulated broken DNA, but in a more discreet range of sizes, particularly with regards to the DSB end corresponding the *oriC* proximal side of the DSB locus (Figure 3B). In contrast to Δ*recB* and Δ*recC* mutants, a significant proportion of DNA isolated from Δ*recD* cells failed to exit the wells of the pulsed-field gels (Figure 3B), indicating the accumulation of branched DNA molecules.

In order to recombine, RecA must be loaded onto the broken DNA ends. Chromosomal binding of RecA was assayed by RecA chromatin immunoprecipitation (ChIP) and whole genome sequencing. In the absence of hairpin cleavage by SbcCD, and irrespective of the presence or absence of RecD, DNA corresponding to a highly expressed gentamycin resistance cassette that was inserted into the *cynX* gene of the strains used in this study (13), was enriched following RecA ChIP (Figure 3C). RecA ChIP was previously shown to result in a general enrichment of highly expressed genes (26) and may be caused by true RecA binding, a bias in crosslinking efficiency (27), or some other unknown reason. In contrast, in response to hairpin cleavage by SbcCD, and again irrespective of the presence (28) or absence of RecD, RecA ChIP-seq revealed RecA binding to the DNA flanking both sides of the DSB locus (Figure 3C and Supplementary Figure S3) indicating that *recD* mutants are capable of loading RecA onto both ends of the DSB at the population level. Given that RecBC and the ATP binding mutant RecBCD^K177Q^ are approximately 10 times less processive than RecBCD *in vitro* (18,29,30), we were surprised that approximately ±20 kb of DNA from the palindrome was enriched for RecA binding in both RecBCD^+^ and Δ*recD* strains (Figure 3C). Unlike RecBCD^+^ cultures, RecA binding was detected between the palindrome and the nearest active Chi sites when RecA ChIP was performed on Δ*recD* cultures (Figure 3C), consistent with the ability of RecBC to load RecA in a Chi-independent manner. There was a close correlation between the peak of binding and the location of the nearest active Chi sequences for both the RecBCD^+^ and Δ*recD* samples (Figure 3C). However, in the case of the *recD* mutant this close correlation was coincidental as the peak of RecA enrichment was not influenced by the position of Chi. It is also noticeable that the amount of RecA bound to the two sides of the DSB is more equal in the Δ*recD* mutant than in the RecBCD+ cells. This may reflect a higher probability, in the presence of fully active RecBCD enzyme, of degrading the origin distal arm of the break all the way to the replication fork converting a two-ended break to a one-ended break. This binding of RecA to the chromosome in response to the induced DSB is sufficient to induce the DNA damage response (Figure 2E).

Following RecA-catalysed strand exchange, homologous recombination in *E. coli* results in the formation of branched 4-strand DNA structures known as Holliday junctions that are resolved by the Holliday junction resolvase complex RuvABC (31). If the cleaved chromosomes of Δ*recD* cells were proceeding to Holliday junction formation, then inactivation of RuvABC (*e.g*. by deletion of the *ruvAB* operon) would result in branched DNA that would fail to exit the wells of gels subject to PFGE (31). Consistent with previous reports and the accumulation of Holliday junctions, DNA isolated from Δ*ruvAB* cells subject to hairpin cleavage by SbcCD showed a reduction in intact, linear DNA and a concomitant increase in branched DNA species (Figure 3D). Compared to DNA isolated form Δ*recD* single mutant cells, there was a notable decrease in the relative amount of both linear intact DNA and broken DNA in DNA isolated form Δ*recD* Δ*ruvAB* cells, with a concomitant relative increase in the amount of branched DNA (Figure 3D). This indicates that in the absence of RecD, DSB ends are processed by homologous recombination and proceed at least to the stage of Holliday junction formation.

### DSB repair in the absence of RecD results in aberrant chromosome amplification

**Figure 4.**
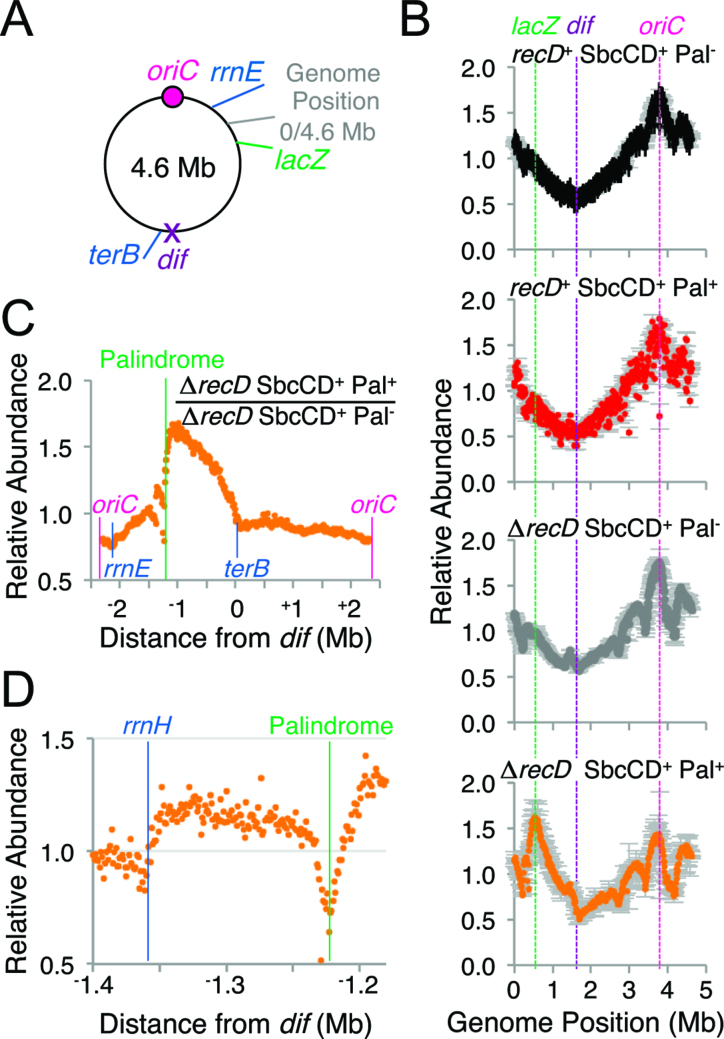
In the absence of RecD, cleavage of the palindrome by SbcCD results in an ectopic origin of chromosomal DNA replication. (**A**) Diagram of the single, circular 4.6 Mb *E. coli* chromosome with relative positions of loci of interest (*oriC, rrnE, lacZ, dif, terB* and the classically defined genomic position 0). (**B**) Marker Frequency Analysis of DNA isolated from *recD*^+^ and Δ*recD* cells containing or not the palindrome (Pal) following 1 h of exponential growth with induced SbcCD expression. Data are the mean of two biological repeats, error bars show the range. Data was averaged using a 10 kb window. (**C** and **D**) DSB-dependent change in relative DNA abundance isolated from Δ*recD* cells. Data values for Δ*recD* SbcCD^+^ Pal^−^ and Δ*recD* SbcCD^+^ Pal^+^ are the average of two biological repeats normalized by the total number of mapped reads. Data was averaged using either a 10 kb window (C), or a 1 kb window (D).

The Δ*recD* specific increase in total cellular DNA content following induction of the DSB (Figure 2D) could be caused by ongoing timely DNA replication initiation at *oriC* in the absence of cellular division and/or aberrant DNA replication initiation events. The DNA replication profile was assayed by marker frequency analysis (MFA) of DNA extracted from asynchronous, exponentially growing cultures of cells. In the absence of hairpin cleavage by SbcCD, and irrespective of the presence or absence of RecD, relative copy number peaked close to the single origin of replication *oriC* and decayed on either side before reaching a minimum close to a sequence known as *dif* that sits approximately half way along the circular chromosome from *oriC* (Figure 4A, B). This observation is consistent with bi-directional replication originating at *oriC* and holds true for DNA extracted from RecBCD^+^ cells undergoing hairpin cleavage by SbcCD (28), Figure 4B. In contrast, DNA extracted from Δ*recD* cells undergoing hairpin cleavage by SbcCD showed a maximal peak that maps close to the DSB locus (Figure 4B).

This DSB-dependent alteration in relative DNA abundance is most clearly visualised by normalising the results obtained for cultures subject to the DSB by the results obtained for an isogenic strain not subject to the DSB (Figure 4C). This analysis showed a DSB-dependent increase in DNA that extends on one side from the palindrome to a sequence known as *terB* in the chromosome terminus and on the other side from the palindrome to the *rrnE* operon, with a dip observed at the *rrnH* operon (Figure 4D). *terB* belongs to family of DNA sequences in the *E. coli* genome that bind Tus protein and blocks DNA replication in a polar manner (12). *rrnH* and *rrnE* are two of the seven heavily transcribed *E. coli* ribosomal RNA operons that are all transcribed in the same direction as DNA replication originating from *oriC*. These results are consistent with a DSB-dependent initiation of replication in Δ*recD* cells that can extend from one side of the break before being blocked by *terB* and on the other side of the break before being blocked first by *rrnH* and subsequently by *rrnE*. This DSB-dependent amplification was not observed in cultures of RecBCD^+^ cells (Figure 4B, Supplementary Figure S4, (28)).

**Figure 5.**
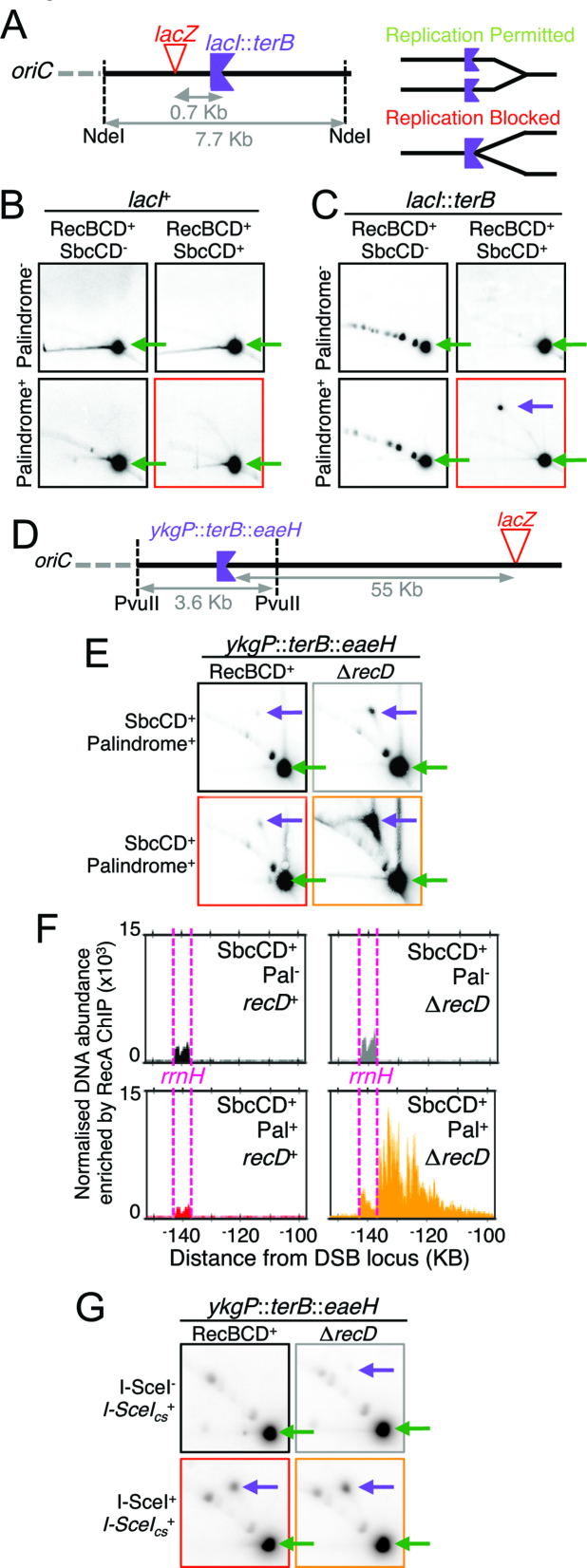
DNA synthesis progressing towards the origin of replication is detected following induction of a DNA double-strand break. (**A**) Diagram showing the location and orientation of *lacI*::*terB*. (**B**) and (**C**) analysis of DNA structure within a 7 Kb fragment containing the palindrome integration site and either an ectopic *terB* sequence (*lacI*::*terB*) oriented to block DNA replication proceeding towards *oriC* (C) or not (B) by 2D gel electrophoresis in RecBCD^+^ cells containing or not the palindrome in *lacZ* following 1h of growth with induced SbcCD expression. (**D**) Diagram showing the location and orientation of *ykgP*::*terB*::*eaeH*. (**E** and **G**) Analysis of DNA structure by 2D gel electrophoresis of a chromosome fragment located 55 Kb *oriC* proximal to *lacZ* and containing an ectopic *terB* site (*ykgP*::*terB*::*eaeH*) that blocks DNA replication proceeding towards *oriC*. (E) DNA isolated from RecBCD^+^ and Δ*recD* cells containing the palindrome in *lacZ* following 1h of growth with SbcCD either expressed (SbcCD^+^) or repressed (SbcCD^−^). (G) DNA isolated from RecBCD^+^ and Δ*recD* cells containing an I-SceI cleavage site (I-SceI_cs_^+^) in *lacZ* following 1h of growth with I-SceI expression either induced (I-SceI^+^) or not (I-SceI^−^). Green arrows indicate the location were linear DNA fragments migrate. Purple arrows indicate the location were branched DNA molecules blocked at *terB* migrate. (**F**) Normalised enrichment of DNA in a 50 kb region containing the *rrnH* locus following RecA chromatin immunoprecipitation (ChIP). Data were both normalized using a median of ratios scaling factor and smoothed using a 1 kb loess filter. Data for all panels is representative from one biological repeat.

DNA replication intermediates can be isolated by 2D gel electrophoresis and the direction of replication determined by integration of an appropriately oriented ectopic *terB* sequence (Figure 5A) (28). 2D gel analysis of a 7.7 kb DNA fragment containing the DSB locus showed a DSB-dependent increase in DNA replication intermediates in RecBCD^+^ cells (Figure 5B). Integration of an ectopic *terB* sequence (oriented to block DNA replication proceeding towards the origin of replication, *oriC*) into this fragment (*lacI*::*terB*, Figure 5A) resulted in accumulation of a specific DNA replication intermediate that was dependent on the presence of a palindrome at *lacZ* (Figure 5C). This indicates that in the presence of RecBCD, cells that have been subject to hairpin cleavage by SbcCD can initiate DNA replication with the origin distal end of the DSB and that this replication can elongate towards *oriC*. 2D gel electrophoresis of DNA isolated from strains with an ectopic *terB* sequence integrated 55 KB upstream of the DSB locus (towards *oriC, ykgP*::*terB*::*eaeH*, Figure 5D) indicated that in a fraction of RecBCD^+^ cells, this replication can elongate at least 55 KB from the DSB site (Figure 5E). Consistent with MFA analysis (Figure 4C), this blocked DNA replication species was substantially more abundant in DNA isolated from Δ*recD* cells subject to the DSB than DNA isolated from RecBCD^+^ cells subject to the DSB (Figure 5E). RecA ChIP of DNA isolated from Δ*recD* cells subject to hairpin cleavage by SbcCD showed a peak of enrichment, *oriC*-distal to the *rrnH* locus that was not observed in RecBCD^+^ cells or Δ*recD* cells not subject to hairpin cleavage by SbcCD (Figure 5F), suggesting that DNA replication forks moving from the palindrome towards *oriC* are blocked at *rrnH* and processed by homologous recombination. Finally, replication intermediates were also detected at *ykgP*::*terB*::*eaeH* progressing towards *oriC* in response to I-SceI induced DSBs (Figure 5G). In contrast to SbcCD-mediated DSBs, this replication was easily detected in DNA isolated from RecBCD^+^ cells and only mildly increased by the absence of RecD. The percentage of radiolabelled probe detected at stalled fork was 2.9 ± 0.1% in DNA extracted from RecBCD^+^ cells compared to 4.3 ± 1.1% in DNA extracted from Δ*recD* cells (*n* = 3, ± standard error of the mean).

## DISCUSSION

Coordination between the two ends of DNA double-strand breaks (DSBs) is critical for their accurate repair. The two ends must invade an appropriate single region of DNA homology. Furthermore, a processive DNA replication fork must not be set up at one end that moves past the location of the other end before it has also been synapsed. Invasion of the second end after the passage of the first replication fork can result in two outwardly facing, divergent forks with the potential to over-replicate part, or all, of the chromosome. In eukaryotic cells, resection of the DNA at the site of a DSB occurs mainly on the 5′ DNA strand leaving the 3′ end close to the site of the break. It seems that coordination of the two ends may involve limited DNA synthesis from the first invading end to extend the D-loop, followed by second-end capture. Second-end capture prevents break-induced replication and promotes gene conversion through the operation of a recombination execution checkpoint that senses the proximity and orientation of the two recombining ends (32). The situation in *E. coli* is different as both the 3′ and 5′ ended DNA strands at the DSB are unwound (and under some conditions degraded) until a Chi site is recognised whereupon a cleaved 3′ ended strand binds RecA and initiates strand invasion and synapsis with the homologous template (16). Given that appropriately oriented Chi sites are likely to be separated from each other by many thousands of base pairs and each recombining end is understood to set up a processive replication fork to restore any missing DNA, it is remarkable that the two ends are well coordinated. How this coordination is achieved is unknown, but we show here that it is deregulated in a *recD* mutant implying that fully functional RecBCD enzyme is required for good coordination of ends. It is interesting to note, given the appearance that prokaryotic and eukaryotic systems might operate differently, that *mec1* (and *mec1 tel1*) mutants of *Saccharomyces cerevisiae*, that are defective for the DNA damage checkpoint, frequently become disomic for chromosome VIII in a reaction suppressed by overexpression of the *DNA2* gene encoding an exonuclease/helicase implicated in end resection during recombination (33). The similarities of the roles of RecBCD and DNA2 in end resection and their proposed roles in limiting over-replication leading to aneuploidy are intriguing.

### *recD* mutants repair and re-cleave DNA at the site of a DNA palindrome

**Figure 6.**
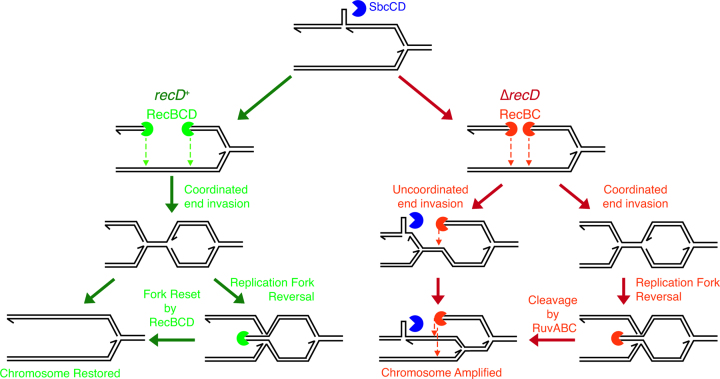
Model for RecBCD-mediated control of DSB-induced DNA synthesis. In the presence of RecBCD, the two ends at the site of a DSB undergo coordinated invasion of the sister chromosome to generate two convergent replication forks that are responsible for the replacement of genetic information lost during end processing. If one of the convergent forks is established before the other and causes the second fork to be reversed, the extruded double-strand end is readily degraded by RecBCD to restore a replication fork. By contrast in the absence of RecD, RecBC either catalyses uncoordinated end invasion or, if end invasion is coordinated, cannot deal with the subsequent fork reversal of one of the convergent forks. Cleavage by RuvABC of the Holliday junction caused by fork reversal that restores one of the ends converting coordinated end invasion to uncoordinated end invasion. In either scenario of uncoordinated end invasion in the absence of RecD, the palindrome is re-cleaved, the chromosome is amplified and viability is lost.

From a genetic perspective a *recD* null mutant is defective for the repair of palindrome-induced DSBs (Figure 1). This is surprising given the observation that in all other assays performed to date *recD* mutants have been found to be recombination proficient (21). However, when we analysed the behaviour of this mutant on pulsed field gels, we observed that the situation was more complicated. DNA fragments, un-cleaved DNA and branched molecules all accumulated in a *recD* mutant, following formation of a palindrome-induced break (Figure 3A). Fragments corresponding to palindrome cleavage could readily be detected as sharp bands by Southern blotting and these accumulated at 60 minutes of SbcCD induction. This was different from the situation observed for a *recB* mutant, where the cleaved DNA fragments were further degraded (presumably by one or more of the numerous nucleases in *E. coli* cells) between 30 and 60 min of induction creating a notably smeared band (13). This, coupled with the observation of little or no loss of the intact un-cleaved fragment and an accumulation of hybridising material in the wells of the gel suggested that repair and re-cleavage were ongoing in the *recD* mutant (Figure 6). That repair is indeed ongoing in the absence of RecD was confirmed by observing a loss of cleaved products and un-cleaved DNA, accompanied by an accumulation of branched molecules in a *recD ruvAB* mutant undergoing DSBR (Figure 3D). This repair and re-cleavage leads to RecA binding to DNA ends (Figure 3C), a stronger SOS response (Figure 2E) and an accumulation of extra DNA per cell that is readily detected by flow cytometry (Figure 2D).

The reason that *recD* mutants re-cleave their chromosomes at the site of a palindrome, whereas *recBCD*^+^ cells do not, is unknown. However, it should be noted that uncoordinated repair is predicted to allow repair forks to pass though the palindrome in one (or other) direction as is the case for normal replication forks that lead to hairpin cleavage by SbcCD. This raises the hypothesis that the lack of palindrome re-cleavage in *recBCD*^+^ cells reflects some aspect of the coordination of DNA ends.

### Aberrant DNA replication is detected in a *recD* mutant undergoing DSBR

MFA of DNA undergoing palindrome-induced DSBR has revealed an accumulation of DNA sequence on both sides of the DSB in a *recD* mutant strain consistent with aberrant replication (Figure 4C). Aberrant replication is confirmed by 2-D gel analysis of chromosomes containing an ectopic *terB* site, providing evidence of DNA replication forks progressing towards the origin of replication at a site 55 Kb origin-proximal of the DSBR event (Figure 5C) that lead to DNA double-strand end formation and RecA binding adjacent to the *rrnH* operon (Figure 5D). It is interesting to note that the accumulation of DNA in a *recD* mutant following attempted DSBR is higher on the origin-distal side of the break than on the origin-proximal side of the break. This suggests a higher frequency of invasion of the origin proximal end than the origin-distal end followed by re-cleavage and re-invasion setting up multiple forks following each other towards the terminus. The reason for this bias towards recombination of the origin-proximal end is currently unknown. However, this is the DNA end that needs to recombine in order to restore a replication fork progressing in the correct direction towards the terminus of the chromosome and the cell may favour this reaction (Figure 6).

### RecA binding reveals extensive unwinding by RecBC enzyme and DSBR at the *rrnH* locus consistent with replication fork breakage

RecA ChIP-seq has revealed the pattern of RecA distribution at a DSB catalysed by RecBC enzyme (Figure 3C). RecA binding rises directly from the site of the double-strand break as expected for an enzyme lacking Chi-regulated exonuclease activity (16). The distribution of RecA binding at the *lacZ* locus where the palindrome-induced DSB is located is surprisingly similar in overall aspect to that observed in a RecBCD^+^ strain, including a correlation of peak DNA enrichment with the nearest active Chi sequences (in this case, an artificial array of three closely spaced Chi sequences (23) integrated 1.5 Kb either side of the DSB locus). This suggests that the unwinding catalysed by RecBC enzyme covers approximately the same genomic region as unwinding by RecBCD enzyme. The Δ*recD* DSB-specific enrichment at the *rrnH* locus (Figure 5D) and corresponding loss of DNA sequence by MFA analysis (Figure 4D) is consistent with recombination following replication fork reversal as was previously shown to occur at inverted *rrn* operons (34).

### RecBCD enzyme co-ordinates the two ends at the site of DSBR

The lack of co-ordination between the two ends of the DSB that we propose to occur at a higher frequency in *recD* mutants (Figure 6), leads to aberrant amplification of the chromosome in a reaction that would lead to three progeny chromosomes from two parental chromosomes if replication were allowed to progress around the genome. However, over-replication extends only as far as the *rrnE* operon on the origin-proximal side of the break and to *terB* on the origin-distal side of the break. This means that the two fully replicated chromosomes are connected via a bridge of DNA extending for up to one half of the length of the genome. This bridge would become extended to twice the length of the entire chromosome by the next rounds of replication from *oriC* if the chromosome did not suffer further cleavage events. However, re-cleavage of the palindrome has been demonstrated during repair (see above) and new rounds of cleavage are expected to be associated with new rounds of replication. These further cleavage events and associated uncoordinated repair events will further interlink the chromosomes in complex ways that will impede accurate segregation. If the chromosomes are to segregate, any bridge DNA will need to be broken at some point along its length leading to chromosomes with partially replicated tails. Alternatively, segregation is blocked and this may be the major contributor to the cell death phenotype observed in the *recD* mutant. Indeed, *recD* cells show clear indication of defective chromosome segregation (Figure 2A and Supplementary Figure S2). Over twenty years ago Tokyo Kogoma and colleagues observed that *recD* mutants exhibited an elevated frequency of induced stable DNA replication (35). Kogoma hypothesised that this was because of un-coordinated DSBR leading to chromosome over-replication (36). This is precisely what we have seen here, except that the initial replication associated with the uncoordinated repair does not extend all the way around the chromosome but is effectively blocked by the *rrnH* operon and by *terB* and that further cleavage and uncoordinated repair events increase the complexity of the chromosomal products.

In Figure 6, we have suggested two stages at which the discoordination of DNA ends may occur. Either the two DNA ends in the *recD* mutant lack some property essential for coordination or one of the ends is regenerated in the *recD* mutant following an initially coordinated reaction. In the first scenario, one end invades before the other because of a missing property of the RecBC enzyme. What this might be is unknown but it is intriguing to note that both monomeric and dimeric forms of RecBCD are evident following purification of the protein (37). Furthermore the RecD2 helicase of *Deinococcus radiodurans* dimerises (38) suggesting that the dimeric form of RecBCD might be mediated via the RecD subunit. Could the dimerization of two RecBCD monomers each engaged with one end mediate their coordination? In the second scenario, the two ends invade in a coordinated manner but one assembles a replisome before the other. As the active replication fork approaches the inactive one, the latter undergoes a replication fork reversal (RFR) reaction. This might be catalysed by positive supercoiling ahead of the active fork (39) or by RuvAB action (40). Following RFR, RuvC cleaves the Holliday junction that has formed and the second end is thus regenerated. This end is now free to recombine with the intact replicated DNA to generate the outward directed replication forks. This is effectively uncoordinated recombination of the two ends albeit mediated via the regeneration of one end following an initially coordinated reaction. In a wild type cell, RecBCD enzyme would rapidly digest the double-strand end generated by RFR, regenerating a replication fork structure and thereby prevent uncoordinated recombination. Further work will be required to distinguish between these possibilities and to understand how RecBCD enzyme ensures the proper coordination of ends.

It surprised us that a *recD* null mutant was defective for DSBR at the site of a chromosomal palindrome when previous assays for recombination by P1 transduction, Hfr mating, bacteriophage lambda *red gam* crosses and sensitivity to UV irradiation indicated no defect or even a hyper-rec phenotype (21). However, it is interesting to note that all of these reactions are effectively one-ended recombination events where the coordination of two ends is clearly not required. By contrast, log-phase cells of a *recD* mutant have been shown to be sensitive to gamma irradiation, which is known to generate two-ended DNA breaks (41). These observations are fully consistent with an important role for RecBCD enzyme in the coordination of two recombining ends. I-SceI cleavage of chromosomes also leads to two-ended DSBs. However, since I-SceI cleaves the DNA in a sequence dependent manner, it can potentially cleave all the sister and cousin chromosomes in a cell as well actively cleave the products of any repair reaction. This complicates the interpretation of any DSBR events observed. In contrast to DNA hairpin cleavage by SbcCD, we detected a loss in viability and a high rate of aberrant DSB-induced chromosomal replication in both RecBCD^+^ and *recD* mutant cells (Figures 1D and 5E). Additionally, RecBCD is maintained at a low copy number per cell (16), and RecD molecules are inactivated following interaction with Chi (42). It is therefore possible that we detected RecBC-mediated reactions in *recD*^+^ cells due to the multitude of DSBs created by I-SceI cleavage. An additional reason for the loss of viability of the *recD* mutant cells undergoing DSBR following palindrome cleavage is likely to be palindrome re-cleavage during repair. This will compound the problems associated with the chromosomal consequences of uncoordinated DSBR by creating further rounds of uncoordinated DSBR.

### General conclusions and perspectives

The coordination of the two DNA ends during DSBR is clearly de-regulated in a *recD* mutant implying that RecBCD enzyme plays an important role in the coordination of ends during recombination in *E. coli*. The consequence of this de-regulation is the amplification of ∼30% of the *E. coli* chromosome. Were the replication forks causing this over-replication not blocked by *ter* sites on one side and oppositely oriented *rrn* operons on the other, replication would run around the chromosome generating three chromosomes from two parents. As it is, the two resulting chromosomes are initially linked by a partially replicated chromosomal section. Further replication forks originating from *oriC* will convert this structure to one where two circular chromosomes are joined by a double chromosome length linker. Re-cleavage of the palindrome and further rounds of uncoordinated repair have the potential to increase the complexity of these chromosomal interconnections. This situation will lead to problems for segregation that are likely to cause chromosome breakage and repair in the next cell cycle or simply to cell death.

Our results demonstrate that chromosome over-replication can occur in the absence of end-coordination during DSBR. In the linear chromosomes of eukaryotic organisms, uncoordinated DSBR has the potential to lead to full aneuploidy if outwardly facing replication forks are allowed to copy an entire chromosome. That this may be possible is attested to by the copying of entire yeast chromosome arms by break-induced replication (10,43). Alternatively, if a partially over-replicated chromosome is inherited this could lead to copy number variation by further processing to incorporate the over-replicated section of DNA into the genome.

## DATA AVAILABILITY

The data files used for chromosome marker frequency analysis have been deposited in the NCBI GEO database under accession number is GSE107973. The data files used for RecA ChIP-seq analysis have been deposited in the NCBI GEO database under accession number GSE107972.

## Supplementary Material

Supplementary Data
