# Supplementary material for: RecBCD coordinates repair of two ends at a DNA double-strand break, preventing aberrant chromosome amplification
Source: Nucleic Acids Res. 2018 Jun 13;46(13):6670–82. doi: 10.1093/nar/gky463 (PMC6061781; doi:10.1093/nar/gky463)
Supplement: Supplementary Data [file gky463_supplemental_files.pdf]

Figure S1. Related to Figure 1

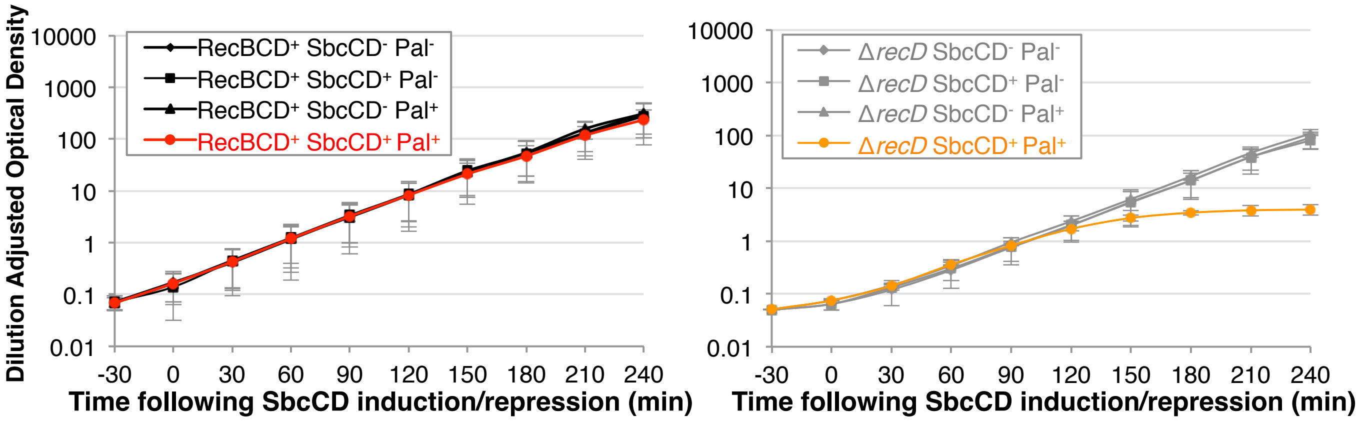

Figure S1. **RecD is required for growth in the presence of an SbcCD-induced DNA double-strand break.** Growth curve showing the effect of SbcCD expression on population growth of strains containing (Pal^{+}) or not (Pal^{-}) the DNA palindrome as measured by optical density of the culture at 600 nm (OD_{600}). SbcCD expression was either induced (SbcCD^{+}) or repressed (SbcCD^{-}) at Time 0 min. Data are represented as mean ± range, n = 2.

Figure S2. Related to Figure 2

Figure S2. Overlay images of cells expressing fluorescent sequence specific (TetR-YFP, LacI-CFP) and non-specific (HupA-mCherry) DNA binding proteins. Cells contain an array of *tetO* sequences and an array of *lacO* sequences either side of *lacZ*. Scale bar shows 5 µm. Data are representative fields of view.

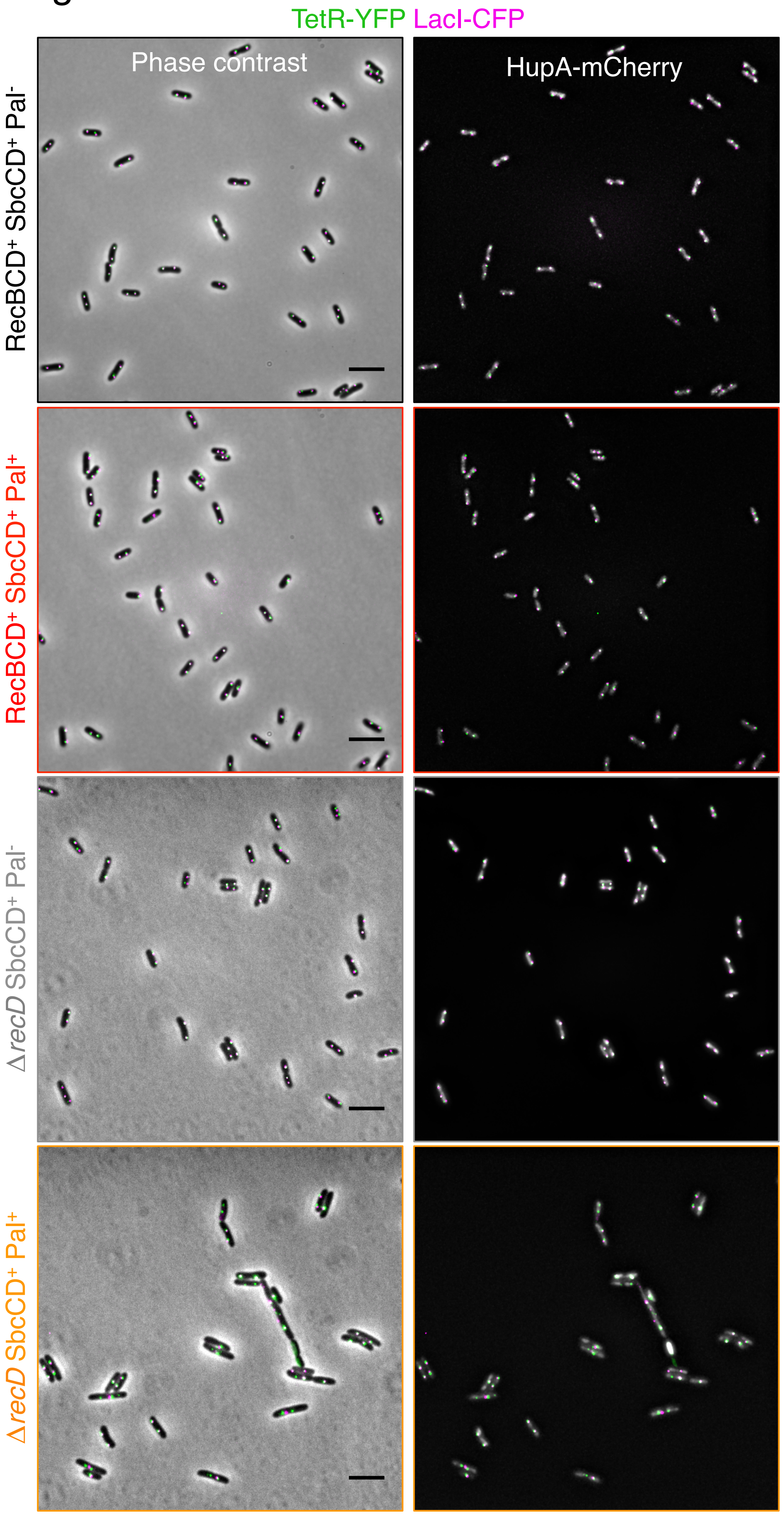

Figure S3. Related to Figure 3

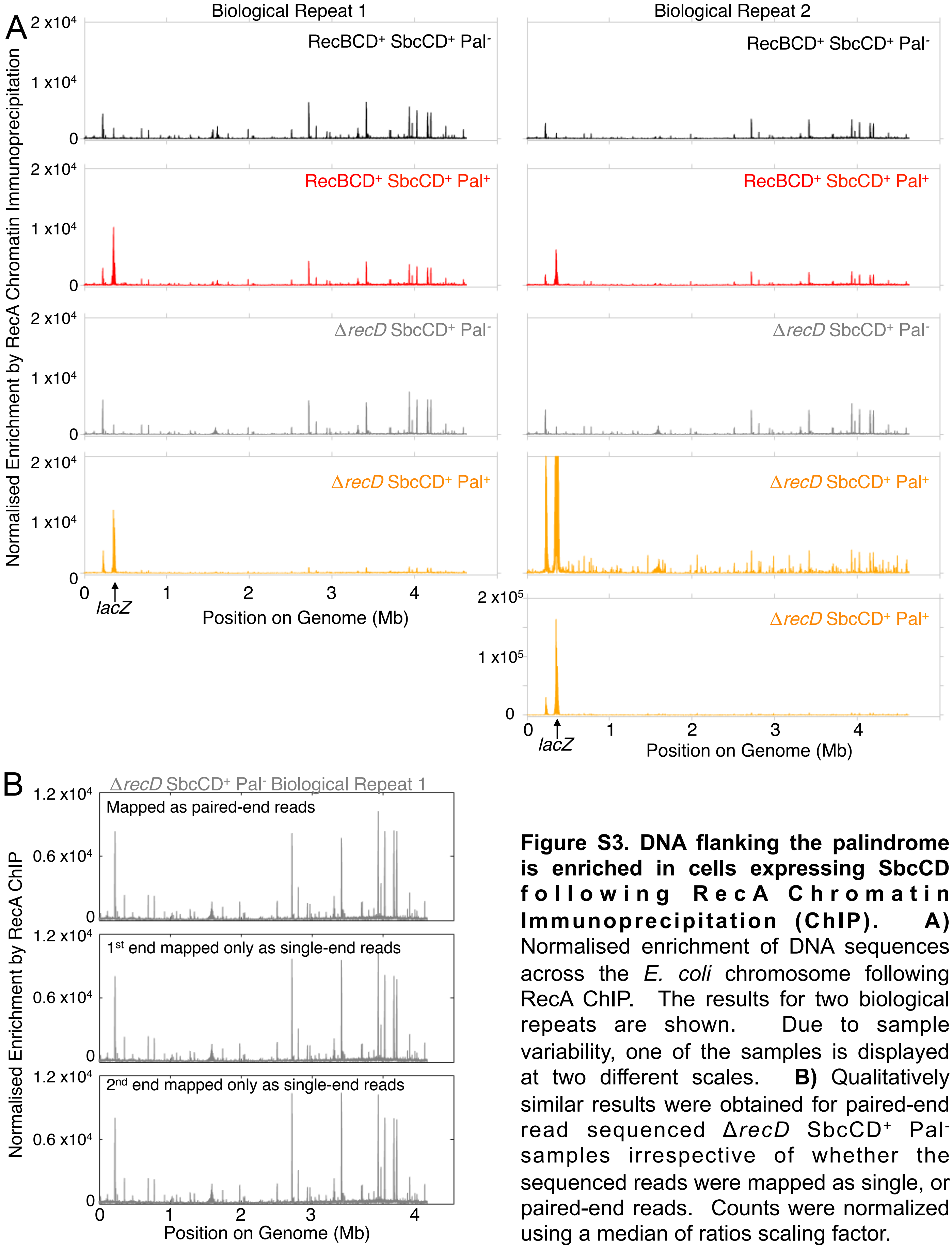

Figure S3. DNA flanking the palindrome is enriched in cells expressing SbcCD following RecA Chromatin Immunoprecipitation (ChIP). A) Normalised enrichment of DNA sequences across the *E. coli* chromosome following RecA ChIP. The results for two biological repeats are shown. Due to sample variability, one of the samples is displayed at two different scales. **B)** Qualitatively similar results were obtained for paired-end read sequenced Δ*recD* SbcCD$^+$ Pal$^-$ samples irrespective of whether the sequenced reads were mapped as single, or paired-end reads. Counts were normalized using a median of ratios scaling factor.

Figure S4. Related to Figure 4

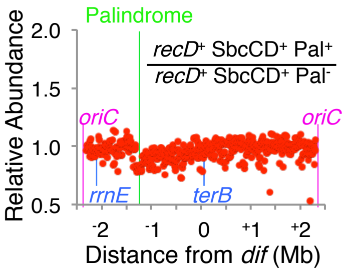

Figure S4. In the presence of RecD, cleavage of the palindrome by SbcCD does not results in an ectopic origin of chromosomal DNA replication. DSB-dependent change in relative DNA abundance isolated from *recD*$^+$ cells. Data values for *recD*$^+$ SbcCD$^+$ Pal$^-$ and *recD*$^+$ SbcCD$^+$ Pal$^+$ are the average of two biological repeats normalized by the total number of mapped reads. Data was averaged using a 10 Kb window.

EXTENDED MATERIALS AND METHODS

Bacterial cell culture and Strain Construction

All bacteria used in this study were derived from the non-pathogenic *E. coli* K12 strain BW27784 (1). For all experiments, cells were grown at 37 °C in LB growth media except those presented in Figures 3F, 3G, S3G and S3H, were cells were grown in M9 minimal media supplemented with glycerol (0.2%) and anhydrotetracycline (1 ng ml^{-1}). Expression of SbcCD and I-SceI was repressed or induced by the addition of glucose or arabinose at the indicated concentration.

Strain Construction

Strains were created by plasmid mediated gene replacement (PMGR), a method for precise modification of *E. coli* chromosomes. Briefly, the parental strain was first transformed using the PMGR vector. Integration of the temperature sensitive plasmid into the host chromosome was selected for by growth at 42 °C in the presence of chloramphenicol (Cm). Subsequent excision of the plasmid was permitted by growth at 30 °C in the absence of Cm, prior to negative selection of the plasmid by growth at 37 °C in the presence of sucrose (5%). Colonies were first screened for Cm sensitivity to ensure plasmid loss, and then screened for the desired modification by Polymerase Chain Reaction (PCR), sequencing and phenotypic tests (when available).

All strains used in this study and the plasmids used for their construction, are listed in Table S1. The following strains were created during the course of this investigation. Strains DL4429, DL4430, DL4474 and DL4475 were created by deletion of *recB* using PMGR vector pDL2698 from strains DL2006, DL2573, DL2792 and DL2793 respectively. Strains DL4436, DL4437, DL4476 and DL4477 were created by deletion of *recC* using PMGR vector pDL4426 from strains DL2006, DL2573, DL2792 and DL2793 respectively. Strains DL3391, DL3743, DL4411, DL4412, DL3816, DL3815, DL5699 and DL6204 were created by deletion of *recD* using PMGR vector pDL2749 from strains DL2006, DL2573, DL2917, DL2988, DL2792, DL2793, DL4184 and DL4201 respectively. Strains DL4196, DL4193, DL4834 and DL4835 were created by deletion of *ruvAB* using PMGR vector pDL2757 from strains DL2792, DL2793, DL3815 and DL3816. Strains DL4848, DL4849, DL4850 and DL4851 were created by insertion of the SOS reporter gene P_{sfiA}-*gfp* into the *attB* locus using PMGR vector pDL4847 from strains DL2006, DL2573, DL3391 and DL3743 respectively. Strains DL4923, DL4924, DL4925, DL4926, DL5098, DL5293 and DL5294 were created by insertion of *terB* between the *ykgP* and *eaeH* genes of DL2006, DL2573, DL3391, DL3743, DL2988, DL2792 and DL2793 respectively using PMGR vector pDL4922. Strain DL5100 was created by first inserting I-$SceI_{csmut4}$ into *lacZ* and subsequently integrating *terB* between the *ykgP* and *eaeH* genes of strain DL2988 using PMGR vectors pDL4996 and pDL4922 respectively. *recD* was deleted from DL5100 using PMGR vector pDL2749 to give strain DL5131. Strains DL4695 and DL4696 were created modifying the chromosomes of DL3276 (2) and DL3277 (2) by consecutive PMGR using PMGR

vectors pDL4068, pDL4522, pDL4680 and pDL4690. Strains DL4708 and DL4709 were created by deletion of *recD* using PMGR vector pDL2749 from strains DL4695 and DL4696 respectively. Strains DL5295 and DL5296 were created by insertion of *terB* into the *lacZ* gene of strains DL2792 and DL2793 respectively using PMGR vector pDL4816.

Plasmid Construction

PMGR vectors are derivatives of the plasmid pTOF24 (3) containing two regions of homology to the *E. coli* chromosome, each approximately 400bp in size. Cloning was carried out using standard molecular biology techniques (PCR, restriction digest, DNA ligation, transformation, and Sanger sequencing). Unless noted otherwise, genomic DNA (gDNA) from *E. coli* strain DL1777 (4) was used as a template for the PCR reactions. All plasmids were maintained in the host *E. coli* strain XL1-Blue (Stratagene).

To create PMGR vector pDL2749, homology arms were synthesized by PCR using primer combinations recD-KO-F1/recD-KO-R1 and recD-KO-F2/recD-KO-R2. The homology arms were fused by crossover PCR using primers recD-KO-F1 and recD-KO-R2 and subsequently cloned into pTOF24 using restriction enzymes PstI and SalI. To create PMGR vector pDL4426, homology arms were synthesized by PCR using primer combinations recC-KO-F1/recC-KO-R1 and recC-KO-F2/recC-KO-R2. The homology arms were fused by crossover PCR using primers recC-KO-F1 and recC-KO-R2 and subsequently cloned into pTOF24 using restriction enzymes PstI and SalI. To create PMGR vector pDL4847, the *attB*::P_{sfiA}-*gfp* gene of strain SS996 (5) was amplified by PCR using primers attB::PsfiA-gfp F and attB::PsfiA-gfp F and cloned into pTOF24 using NotI and SalI restriction enzymes. PMGR vector pDL4996 was created by site-directed mutagenesis of plasmid pDL2521 using primers I-SceIcsSDM4F and I-SceIcsSDM4R. To create PMGR vector pDL4522, the P_{hupA}-*hupA-mCherry* gene of strain SS6322 (6) was amplified by PCR using primers hupA-mCherry_F2 and hupA-mCherry_R2 and cloned into pTOF24 using NotI and XhoI restriction enzymes. To create PMGR vector pDL4816, homology arms were synthesized by PCR using primer combinations 2357/2361 and 2362/2360. The homology arms were fused by crossover PCR using primers 2357 and 2360 and subsequently cloned into pTOF24 using restriction enzymes PstI and SalI. To create PMGR vector pDL2802, homology arms were synthesized by PCR using primer combinations YkgC-F1/ YkgC-R1 and YkgC-F2/ YkgC-R2. The homology arms were fused by crossover PCR using primers YkgC-F1and YkgC-R2 and subsequently cloned into pTOF24 using restriction enzymes PstI and SalI.

PMGR vector pDL4068 was created in a multi-step process. First the P_{ftsKi}-*lacI-cerulean,tetR-eyfp* cassette of pDL3196 was amplified using primers pGB2F and pGB2R and subsequently cloned into plasmid pGB2 (7) using SalI and HindIII restriction enzymes. Next, in order to increase the expression of the *lacI-cerulean,tetR-eyfp* cassette, the promoter P_{ftsKi} was altered by two sequential rounds of site-directed mutagenesis using primer combinations Minus10F/Minus10R and Minus35F/Minus35R to give plasmid pDL4005. This newly created synthetic promoter was named P_{MW1}. The P_{MW1}-*lacI-cerulean,tetR-eyfp* was amplified from

pDL4005 using primers FP_NotI_F and FP_NotI_R2 and subsequently cloned into PMGR vector pDL2802 using NotI restriction enzyme to give pDL4068.

In the course of this investigation it became apparent that recombination could occur due the CFP and YFP genes of the P_{MW1}-*lacI-cerulean,tetR-eyfp* cassette. To prevent this from occurring and improve the signal of *tetO* array bound TetR-YFP we designed an *E. coli* codon optimized *Ypet* gene that shared sequence homology with the *cerulean* gene of no more than 15 bp. This gene was synthesized by Eurofins Genomics and provided as plasmid pCR2.1-Ypet_coli. PMGR vector pDL4680 was created to introduce this *Ypet* cassette into the *E. coli* genome and was created in a multi-step process. First, the *tetR* gene of plasmid pDL3196 was amplified by PCR using primers TetR-mCherryA_F1 and TetR-mCherryA_R1. Next the *Ypet* gene of pCR2.1-Ypet_coli was amplified by PCR using primers tetRYpet_coliF2 and tetRYpet_coliR2. These two PCR products were fused together by crossover PCR using primers TetR-mCherryA_F1 and tetRYpet_coliR2 to create the *tetR-Ypet* gene. The *tetR-eyfp* sequence of pDL4068 was then replaced by *tetR-Ypet* by restriction digest and cloning using XbaI restriction enzyme. The resultant plasmid was named pDL4648. Finally, PMGR vector pDL4680 was created by amplifying *tetR-Ypet-ykgC* from plasmid pDL4648 by PCR using primers YkgC-F1 and pMW11-R and cloning the product into plasmid pTOF24 using PstI and SalI restriction enzymes.

Viability and Growth Tests

Spot tests: SbcCD Sensitivity

Cultures of strains DL2573 (RecBCD$^+$ Pal$^-$), DL2006 (RecBCD$^+$ Pal$^+$), DL4430 (Δ*recB* Pal$^-$), DL4429 (Δ*recB* Pal$^+$), DL4437 (Δ*recC* Pal$^-$), DL4436 (Δ*recC* Pal$^+$), DL3743 (Δ*recD* Pal$^-$) and DL3391 (Δ*recD* Pal$^+$) were grown to late exponential growth phase in LB broth at 37 °C with vigorous shaking. Cultures were then sampled and diluted in LB broth to give a dilution series ranging from OD_{600} 10^{-1} to OD_{600} 10^{-6}. 10 µl of each dilution was then spotted onto an LB agar plate that had been supplemented with either glucose (0.5%) or arabinose (0.2%) to repress (SbcCD$^-$) or induce (SbcCD$^+$) respectively. Petri dishes were incubated overnight at 37 °C prior to image acquisition. Spot tests give a qualitative indication of the ability of cells to divide and form colonies under the indicated growth conditions.

Spot tests: UV-light Sensitivity

Cultures of strains DL2573 (RecBCD$^+$ Palindrome$^-$), DL2006 (RecBCD$^+$ Palindrome$^+$), DL4430 (Δ*recB* Palindrome$^-$), DL4429 (Δ*recB* Palindrome$^+$), DL4437 (Δ*recC* Palindrome$^-$), DL4436 (Δ*recC* Palindrome$^+$), DL3743 (Δ*recD* Palindrome$^-$) and DL3391 (Δ*recD* Palindrome$^+$) were grown, serially diluted, and spotted in replicate onto LB agar plates supplemented with glucose (to repress SbcCD expression) as described above for Spot tests: SbcCD Sensitivity. One petri dish was exposed to 1.2 J/m^2 ultra-violet light, whereas the other was not (0 J/m^2 UV-light). Petri dishes were incubated overnight in the dark at 37 °C prior to image acquisition.

Quantitative Viability Tests

Cultures of SbcCD inducible strains DL2573 (*recD*$^+$ Palindrome$^-$), DL2006 (*recD*$^+$ Palindrome$^+$), DL3743 (*recD*$^-$ Palindrome$^-$), DL3391 (*recD*$^-$ Palindrome$^+$) and I-SceI inducible strains DL2988 (*recD*$^+$ *I-SceI*$_{cs}^-$), DL2917 (*recD*$^+$ *I-SceI*$_{cs}^+$), DL4412 (*recD*$^-$ *I-SceI*$_{cs}^-$), DL4411 (*recD*$^-$ *I-SceI*$_{cs}^+$) were grown to exponential growth phase in LB broth at 37 °C with vigorous shaking. Cultures were diluted to an OD_{600} of 0.1 in either LB broth 0.5% glucose (SbcCD$^-$/I-SceI$^-$)) or LB broth 0.2% arabinose (SbcCD$^+$/I-SceI$^+$). After 1 h of further growth, cultures were sampled, diluted (10^{-4} or 10^{-5}) and plated onto 20 LB agar plates supplemented with 0.5% glucose to repress SbcCD/I-SceI expression. Petri dishes were incubated overnight at 37 °C and the number of colonies counted the next day. The dilution factor for each culture was chosen to give approximately 100 colonies per plate. Three biological repeats were obtained for each strain/condition.

Growth Curves

Overnight (stationary phase) cultures of strains DL2573 (RecBCD$^+$ Pal$^-$), DL2006 (RecBCD$^+$ Pal$^+$), DL3743 (Δ*recD* Pal$^-$) and DL3391 (Δ*recD* Pal$^+$), were diluted to an OD_{600} of 0.05 in LB broth (Time -30 min) and grown at 37 °C with vigorous shaking for 30 min before splitting in two (Time 0) and adding glucose (0.5%) to one to repress SbcCD expression (SbcCD$^-$) and arabinose (0.2%) to the other to induce SbcCD expression (SbcCD$^+$). Cultures were maintained with vigorous shaking at 37 °C and had their OD_{600} monitored every 30 min for 4 h. Cultures were maintained in exponential growth phase by diluting upon reaching an OD_{600} of 0.5. Plotted optical density values are measured OD_{600} corrected for dilution. The results of two biological repeats of each strain are plotted in Figure S1A.

PFGE and Southern Blotting

gDNA Isolation

Cultures of strains DL2792 (RecBCD$^+$ Pal$^-$), DL2793 (RecBCD$^+$ Pal$^+$), DL4474 (Δ*recB* Pal$^-$), DL4475 (Δ*recB* Pal$^+$), DL4476 (Δ*recC* Pal$^-$), DL4477 (Δ*recC* Pal$^+$), DL3816 (Δ*recD* Pal$^-$), DL3815 (Δ*recD* Pal$^+$), DL4196 (Δ*ruvAB* Pal$^-$), DL4193 (Δ*ruvAB* Pal$^+$), DL4835 (Δ*recD* Δ*ruvAB* Pal$^-$) and DL4834 (Δ*recD* Δ*ruvAB* Pal$^+$) were grown to exponential growth phase in LB broth at 37 °C with vigorous shaking. Cultures were diluted to an OD_{600} of 0.02 in either LB broth 0.5% glucose (SbcCD$^-$) or LB broth 0.2% arabinose (SbcCD$^+$). After 1 h of further growth, the optical density of cultures was measured, 10 ml of culture sampled, cells collected by centrifugation, washed twice and concentrated to an OD_{600} of 5 in ice cold TEN buffer (50 mM Tris, 50 mM EDTA, 100 mM NaCl, pH 8.0). Cells were then mixed with an equal volume of 2% low melting point agarose dissolved in TEN buffer and poured into BioRad plug moulds. Once set, plugs were removed from their moulds and incubated overnight in a 1 mg ml^{-1} proteinase K, NDS (0.5

M EDTA, 10 mM Tris, 0.55 M NaOH, 36.8 mM lauryl sarcosine, pH 8.0) solution, at 37 °C with gentle shaking. The following day, plugs were washed 6 times with fresh TE buffer (10 mM Tris, 1 mM EDTA, pH 8.0) with 1 h incubations at room temperature with gentle shaking between washes and stored at 4 °C in TE buffer.

PFGE, Southern Blotting, Probing & Detection

gDNA (in plugs) was washed twice in I-SceI restriction enzyme buffer with 1 h room temperature incubations with gentle shaking between washes. Plugs were then placed in 1 ml of I-SceI restriction enzyme buffer plus I-SceI restriction enzyme and left to digest for 6 h. Digested gDNA was loaded into a 1% agarose in 0.5x TBE (45 mM Tris-borate, 1 mM EDTA) gel and separated using a CHEF-DR II PFGE (Biorad) machine at 6 V/cm for 10 h in 0.5x TBE buffer at 4 °C. Switch time was set to 5 – 30 s with an inclusion angle of 120°. DNA was then transferred to a positively charged nylon membrane by Southern blotting and cross-linked to the membrane using UV-light.

Chromosome fragments of interest were detected using ^{32}P α-dATP incorporated radiolabeled DNA probes that were created using Agilent Prime-It II random labelling kit and a DNA template generated by PCR using either primers DIGperR-F and DIGperR-R (*oriC* proximal probe) or primers DIGmalZ-F and DIGmalZ-R (*oriC* distal probe) and gDNA isolated from strain DL1777 as a template. Probes were hybridized to membranes overnight at 65 °C in 10 ml of Church-Gilbert buffer (7% SDS, 0.5 M NaH_2PO_4, 1 mM EDTA, 1% BSA), then washed twice at 60 °C, first for 15 min in a solution of 0.3 M NaCl, 0.03 M Na citrate and 0.1% SDS, and then for 30 min in a solution of 0.075 M NaCl, 0.0075 M sodium citrate and 0.1% SDS. Radiolabeled membranes were then exposed to a GE healthcare storage phosphor screen. A Molecular Dynamics Storm 860 phosphorImager scanner was used to scan the phospho screen. The resultant image files were visualised using both ImageQuant and FIJI (8) software. Membranes were first probed using one probe and then stripped by incubating for 1 h in a solution composed of 50% formamide, 0.75 M NaCl, 50mM NaH_2PO_4 and 5 mM EDTA at 65 °C, followed by a 30 min incubation in 200 ml of 0.075 M NaCl, 0.0075 M sodium citrate and 0.1% SDS for 30 min at 65 °C, before probing with the second probe. Stripped membranes were exposed and scanned to ensure removal of the original probe.

2D Gel Electrophoresis and Southern Blotting

gDNA Isolation

Cultures of strains DL4924 (RecBCD$^+$ Pal$^-$; Figure 3D), DL4923 (RecBCD$^+$ Pal$^+$; Figure 3D), DL4926 (Δ*recD* Pal$^-$; Figure 3D), DL4925 (Δ*recD* Pal$^+$; Figure 3D), DL5098 (*recD*$^+$ *I-SceI*$_{cs}^-$, Figure 3E; RecBCD$^+$ *I-SceI*$_{cs}^-$ *ykgP*::*terB*::*eaeH*, Figure S3F), DL5100 (*recD*$^+$ *I-SceI*$_{cs}^+$, Figure 3E; RecBCD$^+$ *I-SceI*$_{cs}^+$ *ykgP*::*terB*::*eaeH*, Figure S3F), DL5129 (Δ*recD I-SceI*$_{cs}^-$; Figure 3E), DL5131 (Δ*recD I-SceI*$_{cs}^+$; Figure 3E), DL2792 (RecBCD$^+$ Pal$^-$; Figure S3B), DL2793 (RecBCD$^+$ Pal$^+$; Figure S3B), DL5295 (RecBCD$^+$ Pal$^-$ *lacI*::*terB*; Figure S3C), DL5296 (RecBCD$^+$ Pal$^+$

lacI::*terB*; Figure S3C), DL3391 (Δ*recD* Pal^+; Figure S3D), DL5293 ($RecBCD^+$ Pal^- *ykgP*::*terB*::*eaeH*; Figure S3E), DL5294 ($RecBCD^+$ Pal^+ *ykgP*::*terB*::*eaeH*; Figure S3E), were grown to exponential growth phase in LB broth at 37 °C with vigorous shaking. Cultures were diluted to an OD_{600} of 0.02 in either LB broth 0.5% glucose ($SbcCD^-$/I-$SceI^-$) or LB broth 0.2% arabinose ($SbcCD^+$/I-$SceI^+$). After 1 h of further growth, gDNA was isolated as described above for PFGE ('plug' method), except cells were concentrated to an OD_{600} of 100 in TEN buffer prior to mixing with a 0.8% low melting point agarose in TEN buffer solution.

2D Gel Electrophoresis, Southern Blotting, Probing

Prior to restriction digest, gDNA (in plugs) was washed 6 times in appropriate restriction buffer for 1 h at room temperature with agitation, prior to overnight digestion at 37 °C in restriction buffer with 600 units of restriction enzyme. For analysis of *lacI*::*terB* (Figure S3B, C) gDNA was digested with NdeI restriction enzyme. For analysis of *ykgP*::*terB*::*eaeH* (Figure 3D, E, S3D, E, F) gDNA was digested with PvuII-HF restriction enzyme. Digested DNA was loaded onto a 0.4% agarose gel in 1x TBE (89 mM Tris-borate, 2 mM EDTA) at 1 V/cm for 24 h at 4 °C. Lanes containing the separated DNA were cut out, rotated 90°, and cast in a new gel composed of 1% agarose in 1x TBE supplemented with 0.3 μg ml^{-1} ethidium bromide. This gel (the 2nd dimension) was ran at 6V/cm for 15 h in circulating 1x TBE buffer supplemented with 0.3 μg ml^{-1} ethidium bromide at 4 °C. DNA was then transferred to a positively charged nylon membrane by Southern blotting and cross-linked to the membrane using UV-light. Chromosome fragments of interest were detected using ^{32}P α-dATP incorporated radiolabeled DNA probes as described for PFGE. The probe for detecting the NdeI digested fragment containing (or not) *lacI*::*terB* was prepared using primers lacZp.F and lacZp.R. The probe for detecting the PvuII digested fragment containing (or not) *ykgP*::*terB*::*eaeH* was prepared using primers 3YkgM-F and ykgM-probeR.

Quantification of Blocked Replication Forks

The signal of probed gels was quantified using ImageQuant software. The percentage of DNA stalled at *ykgP*::*terB*::*eaeH* was calculated as 'spot' divided by 'spot' plus 'linear', where 'spot' was the signal within the visible spot corresponding to stalled replication forks (indicated by purple arrow in example images displayed in Figure S3F) minus the average background signal and 'linear' was the signal within the visible spot corresponding to linear DNA (indicated by green arrow in example images displayed in Figure S3F) minus the average background signal. Three biological repeats were quantified.

Chromosome Marker Frequency Analysis

Genomic DNA Isolation and Illumina Sequencing

Cultures of strains DL2573 (*recD*$^+$ Pal$^+$), DL2006 (*recD*$^+$ Pal$^+$), DL3743 (Δ*recD* Pal$^-$) and DL3391 (Δ*recD* Pal$^+$) were grown to exponential growth phase in LB broth at 37 °C prior to inducing SbcCD expression by the addition of arabinose (final concentration 0.2%). After 1 h of growth in SbcCD$^+$ conditions, gDNA was isolated from 20 ml samples of these cultures using a Wizard Genomic DNA Purification Kit following manufacturer's guidelines. Purified gDNA was treated with the supplied RNase for 50 min and rehydrated overnight in the supplied TE buffer at 4 °C. Three units of the RNase blend Riboshredder was then added to further destroy contaminating RNA. Samples were then purified again by phenol/chloroform extraction and ethanol precipitation. In tandem, gDNA was isolated using the same procedure form non-replicating stationary phase cultures of strain DL2573 to act as a control for sequence bias. Libraries were prepared from the gDNA by Edinburgh Genomics using an Illumina TruSeq DNA Sample Prep kit. Edinburgh Genomics subsequently obtained paired-end reads of the samples using an Illumina HiSeq 2000 platform. Two biological repeats of each strain were acquired.

Marker Frequency Analysis

Edinburgh Genomics supplied paired-end reads with adapter sequences removed. Reads were aligned to the DL2573 draft reference genome sequence (GSE107973) using the Burrows-Wheels Alignment software BWA-MEM and the number of reads mapped to each bp of the genome quantified using SAMtools (mpileup). Technical repeats (multiple sequencing runs of the same biological gDNA library) were combined by merging BAM files using SAMtools software (9) in order to improve coverage. Mapped reads was averaged over 1Kb or 10Kb bins (as indicated in Figure legends) using the software R.

RecA ChIP-seq

Chromatin ImmunoPrecipitation

Cultures of strains DL4201 (RecBCD$^+$ Pal$^-$), DL4184 (RecBCD$^+$ Pal$^+$), DL6204 (Δ*recD* Pal$^-$) and DL5699 (Δ*recD* Pal$^+$) were grown to exponential growth phase in LB broth supplemented with glucose (final concentration of 0.5%) at 37 °C prior to inducing SbcCD expression by the addition of arabinose (final concentration 0.2%). After 1 h of growth in SbcCD$^+$ conditions, cells were fixed by the addition of formaldehyde (final concentration 1%) for 10 min at 22.5 °C to crosslink proteins to DNA. Crosslinking was quenched by the addition of 0.5 M glycine. Cells were then collected by centrifugation at 1,500 x g for 7 min before washing three times in ice-cold 1x PBS and re-suspending in 250 µl of ChIP buffer (10 ml ChIP buffer consists of 200 mM Tris-HCl (pH 8.0), 600 mM NaCl 4% Triton X and 1 cOmplete™ protease inhibitor cocktail EDTA-free tablet). Samples were then sonicated using a Diagenode Bioruptor® at 30 seconds intervals for 10 minutes at high amplitude. After sonication, 350 µl of ChIP buffer was added to each sample and the samples gently mixed by pipetting. Immunoprecipitation was performed overnight at 4°C using 1/100 anti-RecA antibody (Abcam, ab63797). Immunoprecipitated samples were then incubated with Protein G Dynabeads® for 2 hours with rotation at room

temperature. All samples were washed three times with 1 X PBS + 0.02% Tween-20 before re-suspending the Protein G Dynabeads® in 200 µl of TE buffer + 1% SDS. 100 µl of TE buffer + 1% SDS were added to the input samples and all samples were then incubated at 65°C for 10 hours to reverse the formaldehyde cross-links. DNA was isolated using the MinElute PCR purification kit according to manufacturer's instructions. DNA was eluted in 100 µl of TE buffer using a 2-step elution. Samples were stored at -20°C. Two biological repeats of each strain were acquired.

Illumina ChIP-seq Library Preparation

Libraries of the immunoprecipitated DNA were made using NEBNext® ChIP-Seq library preparation kit. Briefly, the samples were first subjected to end repair to fill in ssDNA overhangs, remove 3' phosphates and phosphorylate the 5' ends of DNA. Klenow exo- was used to adenylate the 3' ends of the DNA and NEBNext DNA adaptors (provided in the NEBNext Multiplex Oligos for Illumina kit) were ligated using T4 DNA ligase. After each step, the DNA was purified using the Qiagen MinElute PCR purification kit according to the manufacturer's instructions. After adaptor ligation, the adaptor-modified DNA fragments were enriched by PCR using primers (provided in the NEBNext Multiplex Oligos for Illumina kit) corresponding to the beginning of each adaptor. Finally, agarose gel electrophoresis was used to size select adaptor-ligated DNA with an average size of approximately 300 bp. All samples were quantified on a Bioanalyzer (Agilent) before being sequenced on either an Illumina® HiSeq 2500 (for DL4184, DL4201 and DL5699) or HiSeq 4000 (for DL6204) by Edinburgh Genomics.

ChIP-seq Data Analysis

50 bp single-end reads (for DL4184, DL4201 and DL5699) and 75 bp pair-end reads (for DL6204) were mapped to the DL4201 draft reference genome sequence (GSE107972) using the default parameters of software Bowtie 2 (10). As a control, the two sequenced ends for DL6204 samples were mapped individually as single-end reads (Figure S2B). Read depths were calculated using SAMtools software (with parameter –d set to 10^6). A single, sample-specific scaling factor was applied to the number of mapped reads for each position of the genome to normalise for differences in sequencing depth. To calculate this scaling factor, we implemented the median of ratios normalisation of DESeq software (11) using R. Operationally this involved dividing the number of mapped reads for each genomic position of the sample of interest by the geometric mean of the number of mapped reads for the corresponding genomic position across all eight samples (two biological repeats of four strains). The scaling factor was the median value for all genomic positions. This normalisation is based on the hypothesis that the perturbations (DSB induction and absence of *recD*) alter RecA binding to chromosomal DNA at a minority (<50%) of gDNA positions along the chromosome relative to controls. This hypothesis was deemed reasonable as the effect of the DSB on gDNA enrichment following RecA ChIP was only seen across <3% of the genome and the region amplified (as detected by MFA) was <30% of the genome. Following this normalization, values were smoothed using the

MATLAB loess local regression function as documented in the Figure legends. The conclusions that were drawn in this paper are neither dependent upon the specific normalization method or binning method used as the same qualitative effects are observed using the number of mapped reads prior to normalization and binning.

Flow Cytometry

P_{sfiA}-gfp Measurements

Cultures of strains DL4849 (RecBCD$^+$ Pal$^-$), DL4848 (RecBCD$^+$ Pal$^+$), DL4851 (Δ*recD* Pal$^-$) and DL4850 (Δ*recD* Pal$^+$) were grown to exponential growth phase in LB broth at 37 °C with vigorous shaking. Cultures were diluted to an OD_{600} of 0.005 in either LB broth 0.5% glucose (SbcCD$^-$) or LB broth 0.5% glucose (SbcCD$^+$). After 2 h of further growth, cultures were sampled and GFP fluorescence measured using an Apogee A50 flow cytometer. Data were saved as .csv files and analysed using MATLAB software. Either two or three biological repeats of each strain and condition were acquired.

Relative Cellular DNA content measurements

Cultures of strains DL2573 (RecBCD$^+$ Pal$^-$), DL2006 (RecBCD$^+$ Pal$^+$), DL3743 (Δ*recD* Pal$^-$) and DL3391 (Δ*recD* Pal$^+$) were grown to exponential growth phase in LB broth at 37 °C with vigorous shaking. Cultures were diluted to an OD_{600} of 0.02 in either LB broth 0.5% glucose (SbcCD$^-$) or LB broth 0.2% arabinose (SbcCD$^+$). After 1 h of further growth, cells were fixed by adding 1 ml of culture to 8 ml of 100% ethanol. Fixed cells were collected by centrifugation, washed twice in 1x PBS and re-suspended in 400 µl of 1x PBS. RNA was then degraded and DNA stained by the addition of 100 µl of a 1x PBS, 50 µg ml^{-1} propidium iodide, 500 µl ml^{-1} RNaseA solution. Propidium iodide fluorescence (a measure of DNA content) was measured using an Apogee A50 flow cytometer. Data were saved as .csv files and analysed using MATLAB software. Three biological repeats of each strain and condition were acquired.

Microscopy

Cultures of strains DL4696 (RecBCD$^+$ Pal$^-$), DL4695 (RecBCD$^+$ Pal$^+$), DL4709 (Δ*recD* Pal$^-$) and DL4708 (Δ*recD* Pal$^+$) were grown to exponential growth phase in M9 minimal growth media supplemented with 0.2% glycerol and 1 ng ml^{-1} anhydrotetracycline at 37 °C with vigorous shaking to an OD_{600} of 0.2 after which either SbcCD was induced (SbcCD$^+$) or repressed (SbcCD$^-$) by the addition of arabinose (0.2%) or glucose (0.2%) respectively. After 1 h of further growth, 10 µl of cell culture was mounted on a pad of 1% agarose.H_2O, covered with #1.5 coverslip and imaged by widefield fluorescence microscopy at a resolution of 100nm X, 100nm Y, 200nm Z using a Zeiss Axiovert 200 fluorescence microscope equipped with a 100x Objective NA1.4 phase objective with a 1.6x Optivar, Photometrics Evolve 512 EMCCD camera, Xenon light source and piezo stage. The microscope was controlled using Metamorph software.

Acquired images were deconvolved using Autoquant X2 and visualized and processed using FIJI. Three biological repeats were acquired for each strain.

Table S1

REAGENT or RESOURCE	SOURCE	IDENTIFIER
**Deposited Data**		
DL2573 chromosome marker frequency analysis Exponential Growth Biological Repeat 1	This paper	NCBI GEO; GSM2884560
DL2573 chromosome marker frequency analysis Exponential Growth Biological Repeat 2	This paper	NCBI GEO; GSM2884561
DL2006 chromosome marker frequency analysis Exponential Growth Biological Repeat 1	This paper	NCBI GEO; GSM2884558
DL2006 chromosome marker frequency analysis Exponential Growth Biological Repeat 2	This paper	NCBI GEO; GSM2884559
DL3391 chromosome marker frequency analysis Exponential Growth Biological Repeat 1	This paper	NCBI GEO; GSM2884562
DL3391 chromosome marker frequency analysis Exponential Growth Biological Repeat 2	This paper	NCBI GEO; GSM2884563
DL3743 chromosome marker frequency analysis Exponential Growth Biological Repeat 1	This paper	NCBI GEO; GSM2884564
DL3743 chromosome marker frequency analysis Exponential Growth Biological Repeat 2	This paper	NCBI GEO; GSM2884565
DL2573 chromosome marker frequency analysis Stationary Growth Biological Repeat 1	This paper	NCBI GEO; GSM2884566
DL2573 chromosome marker frequency analysis Stationary Growth Biological Repeat 2	This paper	NCBI GEO; GSM2884567
DL4184 RecA ChIP-seq Biological Repeat 1	This paper	NCBI GEO; GSM2884550
DL4184 RecA ChIP-seq Biological Repeat 2	(12)	NCBI GEO; GSM2884551 Reanalysis of GSM2045608
DL4201 RecA ChIP-seq Biological Repeat 1	This paper	NCBI GEO; GSM2884552
DL4201 RecA ChIP-seq Biological Repeat 2	(12)	NCBI GEO; GSM2884553 Reanalysis of GSM2045609
DL5699 RecA ChIP-seq Biological Repeat 1	This paper	NCBI GEO; GSM2884554
DL5699 RecA ChIP-seq Biological Repeat 2	This paper	NCBI GEO; GSM2884555
DL6204 RecA ChIP-seq Biological Repeat 1	This paper	NCBI GEO; GSM2884556
DL6204 RecA ChIP-seq Biological Repeat 2	This paper	NCBI GEO; GSM2884557

**Strains**		
E. coli; *endA1 gyrA96(nal^R) thi-1 recA1 relA1 lac glnV44 F'[::Tn10 proAB$^+$ lacI^q Δ(lacZ)M15] hsdR17(r$_K^-$ m$_K^+$)*	Stratagene	XL1 Blue
E. coli K12; MG1655 *lacI^q lacZχ- fnr-267*	(4)	DL1777
E. coli K12; MG1655 *lacI^q rrnB3* Δ*lacZ4787 hsdR514* Δ(*araBAD*)*567* Δ(*rhaBAD*)*568* Δ(*araFGH*) Φ (ΔP$_{araE}$ P$_{CP18}$-*araE*) *ydeV*::*IS1 valUX$^-$*	(1)	BW27784
E. coli; JC13509 P$_{hupA}$-*hupA-mCherry,frt*	(6)	SS6322
E. coli; Δ*attB*::P$_{sfiA}$-*gfp thi-1 argE3 his-4*	(5)	SS996
P$_{BAD}$-*sbcDC lacZ*::*pal246 cynX*::*[(240xtetO)::Gm^R] mhpC*::*[(240xlacO)*::*Km^R]* Δ*lacI lacZχ- mhpA*::*χχχ lacZ*::*χχχ*::*lacY*	(2)	DL3276
P$_{BAD}$-*sbcDC lacZ$^+$ cynX*::*[(240xtetO)::Gm^R] mhpC*::*[(240xlacO)*::*Km^R]* Δ*lacI lacZχ- mhpA*::*χχχ lacZ*::*χχχ*::*lacY*	(2)	DL3277
E. coli K12; BW27784 P$_{BAD}$-*sbcDC lacZ::pal246 cynX::Gm^R lacI^q lacZχ-*	(4)	DL2006
E. coli K12; BW27784 P$_{BAD}$-*sbcDC lacZ$^+$ cynX::Gm^R lacI^q lacZχ-*	(4)	DL2573
E. coli K12; BW27784 Δ*recB* P$_{BAD}$-*sbcDC lacZ::pal246 cynX::Gm^R lacI^q lacZχ-*	This paper	DL4429
E. coli K12; BW27784 Δ*recB* P$_{BAD}$-*sbcDC lacZ$^+$ cynX::Gm^R lacI^q lacZχ-*	This paper	DL4430
E. coli K12; BW27784 Δ*recC* P$_{BAD}$-*sbcDC lacZ::pal246 cynX::Gm^R lacI^q lacZχ-*	This paper	DL4436
E. coli K12; BW27784 Δ*recC* P$_{BAD}$-*sbcDC lacZ$^+$ cynX::Gm^R lacI^q lacZχ-*	This paper	DL4437
E. coli K12; BW27784 Δ*recD* P$_{BAD}$-*sbcDC lacZ::pal246 cynX::Gm^R lacI^q lacZχ-*	This paper	DL3391
E. coli K12; BW27784 Δ*recD* P$_{BAD}$-*sbcDC lacZ$^+$ cynX::Gm^R lacI^q lacZχ-*	This paper	DL3743
E. coli K12; BW27784 *lacZ::I-SceI$_{cs}$ araB::P$_{BAD}$-I-SceI lacI^q lacZχ-*	This paper	DL2917
E. coli K12; BW27784 *araB::P$_{BAD}$-I-SceI lacI^q lacZχ-*	This paper	DL2988
E. coli K12; BW27784 Δ*recD lacZ::I-SceI$_{cs}$ araB::P$_{BAD}$-I-SceI lacI^q lacZχ-*	This paper	DL4411
E. coli K12; BW27784 Δ*recD araB::P$_{BAD}$-I-SceI lacI^q lacZχ-*	This paper	DL4412
E. coli K12; BW27784 *lacZ::ISceI$_{cs}$ proA::ISceI$_{cs}$ tsx::ISceI$_{cs}$* P$_{BAD}$-*sbcDC cynX::Gm^R lacI^q lacZχ-*	(4)	DL2849
E. coli K12; BW27784 *proA::ISceI$_{cs}$ tsx::ISceI$_{cs}$* P$_{BAD}$-*sbcDC lacZ$^+$ cynX::Gm^R lacI^q lacZχ-*	(4)	DL2792
E. coli K12; BW27784 *proA::ISceI$_{cs}$ tsx::ISceI$_{cs}$* P$_{BAD}$-*sbcDC lacZ::pal246 cynX::Gm^R lacI^q lacZχ-*	(4)	DL2793
E. coli K12; BW27784 Δ*recB proA::ISceI$_{cs}$ tsx::ISceI$_{cs}$* P$_{BAD}$-*sbcDC lacZ$^+$ cynX::Gm^R lacI^q lacZχ-*	This paper	DL4474

E. coli K12; BW27784 ΔrecB proA::ISceI$_{cs}$ tsx::ISceI$_{cs}$ P$_{BAD}$-sbcDC lacZ::pal246 cynX::Gm^{R} lacI^{q} lacZχ-	This paper	DL4475
E. coli K12; BW27784 ΔrecC proA::ISceI$_{cs}$ tsx::ISceI$_{cs}$ P$_{BAD}$-sbcDC lacZ^{+} cynX::Gm^{R} lacI^{q} lacZχ-	This paper	DL4476
E. coli K12; BW27784 ΔrecC proA::ISceI$_{cs}$ tsx::ISceI$_{cs}$ P$_{BAD}$-sbcDC lacZ::pal246 cynX::Gm^{R} lacI^{q} lacZχ-	This paper	DL4477
E. coli K12; BW27784 ΔrecD proA::ISceI$_{cs}$ tsx::ISceI$_{cs}$ P$_{BAD}$-sbcDC lacZ^{+} cynX::Gm^{R} lacI^{q} lacZχ-	This paper	DL3816
E. coli K12; BW27784 ΔrecD proA::ISceI$_{cs}$ tsx::ISceI$_{cs}$ P$_{BAD}$-sbcDC lacZ::pal246 cynX::Gm^{R} lacI^{q} lacZχ-	This paper	DL3815
E. coli K12; BW27784 ΔruvAB proA::ISceI$_{cs}$ tsx::ISceI$_{cs}$ P$_{BAD}$-sbcDC lacZ^{+} cynX::Gm^{R} lacI^{q} lacZχ-	This paper	DL4196
E. coli K12; BW27784 ΔruvAB proA::ISceI$_{cs}$ tsx::ISceI$_{cs}$ P$_{BAD}$-sbcDC lacZ::pal246 cynX::Gm^{R} lacI^{q} lacZχ-	This paper	DL4193
E. coli K12; BW27784 ΔruvAB ΔrecD proA::ISceI$_{cs}$ tsx::ISceI$_{cs}$ P$_{BAD}$-sbcDC lacZ::pal246 cynX::Gm^{R} lacI^{q} lacZχ-	This paper	DL4834
E. coli K12; BW27784 ΔruvAB ΔrecD proA::ISceI$_{cs}$ tsx::ISceI$_{cs}$ P$_{BAD}$-sbcDC lacZ^{+} cynX::Gm^{R} lacI^{q} lacZχ-	This paper	DL4835
E. coli K12; BW27784 mhpR::χχχ lacZ::χχχ proA::ISceI$_{cs}$ tsx::ISceI$_{cs}$ P$_{BAD}$-sbcDC lacZ::pal246 cynX::Gm^{R} lacI^{q} lacZχ-	(13)	DL4184
E. coli K12; BW27784 mhpR::χχχ lacZ::χχχ proA::ISceI$_{cs}$ tsx::ISceI$_{cs}$ P$_{BAD}$-sbcDC lacZ^{+} cynX::Gm^{R} lacI^{q} lacZχ-	(13)	DL4201
E. coli K12; BW27784 ΔrecD mhpR::χχχ lacZ::χχχ proA::ISceI$_{cs}$ tsx::ISceI$_{cs}$ P$_{BAD}$-sbcDC lacZ::pal246 cynX::Gm^{R} lacI^{q} lacZχ-	This paper	DL5699
E. coli K12; BW27784 ΔrecD mhpR::χχχ lacZ::χχχ proA::ISceI$_{cs}$ tsx::ISceI$_{cs}$ P$_{BAD}$-sbcDC lacZ^{+} cynX::Gm^{R} lacI^{q} lacZχ-	This paper	DL6204
E. coli K12; BW27784 ΔattB::P$_{sfiA}$-gfp P$_{BAD}$-sbcDC lacZ::pal246 cynX::Gm^{R} lacI^{q} lacZχ-	This paper	DL4848
E. coli K12; BW27784 ΔattB::P$_{sfiA}$-gfp P$_{BAD}$-sbcDC lacZ^{+} cynX::Gm^{R} lacI^{q} lacZχ-	This paper	DL4849
E. coli K12; BW27784 ΔattB::P$_{sfiA}$-gfp ΔrecD P$_{BAD}$-sbcDC lacZ::pal246 cynX::Gm^{R} lacI^{q} lacZχ-	This paper	DL4850
E. coli K12; BW27784 ΔattB::P$_{sfiA}$-gfp ΔrecD P$_{BAD}$-sbcDC lacZ^{+} cynX::Gm^{R} lacI^{q} lacZχ-	This paper	DL4851
E. coli K12; BW27784 ykgP::terB::eaeH P$_{BAD}$-sbcDC lacZ::pal246 cynX::Gm^{R} lacI^{q} lacZχ-	This paper	DL4923
E. coli K12; BW27784 ykgP::terB::eaeH P$_{BAD}$-sbcDC lacZ^{+} cynX::Gm^{R} lacI^{q} lacZχ-	This paper	DL4924
E. coli K12; BW27784 ykgP::terB::eaeH ΔrecD P$_{BAD}$-sbcDC lacZ::pal246 cynX::Gm^{R} lacI^{q} lacZχ-	This paper	DL4925
E. coli K12; BW27784 ykgP::terB::eaeH ΔrecD P$_{BAD}$-sbcDC lacZ^{+} cynX::Gm^{R} lacI^{q} lacZχ-	This paper	DL4926

E. coli K12; BW27784 *ykgP*::*terB*::*eaeH araB*::P_{BAD}-*I-SceI lacI*^{q} *lacZχ-*	This paper	DL5098
E. coli K12; BW27784 *ykgP*::*terB*::*eaeH lacZ*::*I-SceI*$_{csmut4}$ *araB*::P_{BAD}-*I-SceI lacI*^{q} *lacZχ-*	This paper	DL5100
E. coli K12; BW27784 Δ*recD ykgP*::*terB*::*eaeH araB*::P_{BAD}-*I-SceI lacI*^{q} *lacZχ-*	This paper	DL5129
E. coli K12; BW27784 Δ*recD ykgP*::*terB*::*eaeH lacZ*::*I-SceI*$_{csmut4}$ *araB*::P_{BAD}-*I-SceI lacI*^{q} *lacZχ-*	This paper	DL5131
E. coli K12; BW27784 *rph*$^{+}$ *ykgC*::P_{mw1}-*lacI-cerulean,tetR-Ypet hupA-mCherry* P_{BAD}-*sbcDC lacZ*::*pal246 cynX*::*[(240xtetO)*::*Gm*^{R}*]* *mhpC*::*[(240xlacO)*::*Km*^{R}*]* Δ*lacI lacZχ- mhpA*::*χχχ lacZ*::*χχχ*::*lacY*	This paper	DL4695
E. coli K12; BW27784 *rph*$^{+}$ *ykgC*::P_{mw1}-*lacI-cerulean,tetR-Ypet hupA-mCherry* P_{BAD}-*sbcDC lacZ*$^{+}$ *cynX*::*[(240xtetO)*::*Gm*^{R}*]* *mhpC*::*[(240xlacO)*::*Km*^{R}*]* Δ*lacI lacZχ- mhpA*::*χχχ lacZ*::*χχχ*::*lacY*	This paper	DL4696
E. coli K12; BW27784 Δ*recD rph*$^{+}$ *ykgC*::P_{mw1}-*lacI-cerulean,tetR-Ypet hupA-mCherry* P_{BAD}-*sbcDC lacZ*::*pal246 cynX*::*[(240xtetO)*::*Gm*^{R}*]* *mhpC*::*[(240xlacO)*::*Km*^{R}*]* Δ*lacI lacZχ- mhpA*::*χχχ lacZ*::*χχχ*::*lacY*	This paper	DL4708
E. coli K12; BW27784 Δ*recD rph*$^{+}$ *ykgC*::P_{mw1}-*lacI-cerulean,tetR-Ypet hupA-mCherry* P_{BAD}-*sbcDC lacZ*$^{+}$ *cynX*::*[(240xtetO)*::*Gm*^{R}*]* *mhpC*::*[(240xlacO)*::*Km*^{R}*]* Δ*lacI lacZχ- mhpA*::*χχχ lacZ*::*χχχ*::*lacY*	This paper	DL4709
E. coli K12; BW27784 *lacI*::*terB proA*::*ISceI*$_{cs}$ *tsx*::*ISceI*$_{cs}$ P_{BAD}-*sbcDC lacZ*$^{+}$ *cynX*::*Gm*^{R} *lacI*^{q} *lacZχ-*	This paper	DL5295
E. coli K12; BW27784 *lacI*::*terB proA*::*ISceI*$_{cs}$ *tsx*::*ISceI*$_{cs}$ P_{BAD}-*sbcDC lacZ*::*pal246 cynX*::*Gm*^{R} *lacI*^{q} *lacZχ-*	This paper	DL5296
E. coli K12; BW27784 *ykgP*::*terB*::*eaeH proA*::*ISceI*$_{cs}$ *tsx*::*ISceI*$_{cs}$ P_{BAD}-*sbcDC lacZ*$^{+}$ *cynX*::*Gm*^{R} *lacI*^{q} *lacZχ-*	This paper	DL5293
E. coli K12; BW27784 *ykgP*::*terB*::*eaeH proA*::*ISceI*$_{cs}$ *tsx*::*ISceI*$_{cs}$ P_{BAD}-*sbcDC lacZ*::*pal246 cynX*::*Gm*^{R} *lacI*^{q} *lacZχ-*	This paper	DL5294
**Plasmids**		
pDL3196; Amp^{R} P_{ftsKi}-*lacI-cerulean,tetR-eyfp*	(2)	pDL3196
pGB2; Spc^{R}	(7)	pGB2
pMW5; Spc^{R} P_{MW1}-*lacI-cerulean,tetR-eyfp*	This paper	pDL4005
pTOF24; *repA*^{TS} *sacB* Cm^{R} Km^{R}	(3)	pTOF24
pTOF-*lacZ*::*I-SceI*	(4)	pDL2521
pTOF-Δ*recB*	(14)	pDL2698
pTOF-Δ*recC*	This paper	pDL4426
pTOF-Δ*recD*	This paper	pDL2749
pTOF-Δ*ruvAB*	(13)	pDL2757
pTOF-*ykgC*::NotI	This paper	pDL2802

pTOF-Δ*attB*::P$_{sfiA}$-*gfp*	This paper	pDL4847
pTOF-*ykgP*::*terB*::*eaeH*	(12)	pDL4922
pTOF-*lacZ*::*I-SceI*$_{csmut4}$	This paper	pDL4996
pTOF-*lacI*::*terB*	This paper	pDL4816
pTOF-*ykgC*::P$_{mw1}$-*lacI-cerulean,tetR-eyfp*	This paper	pDL4068
pTOF-*hupA-mCherry*	This paper	pDL4522
pCR2.1-Ypet_coli;	Eurofins Genomics; This paper	pDL4638
pTOF-*ykgC*::P$_{mw1}$-*lacI-cerulean,tetR-Ypet*	This paper	pDL4648
pTOF-*tetR-Ypet-ykgC*	This paper	pDL4680
pTOF-*rph*$^{+}$	(15)	pDL4690
**Oligonucleotides**		
AAAAACTGCAGAGGCGAAGTCATTGATGGAA	Eurofins Genomics; This paper	recC-KO-F1
AACAGCGGTAACAGGAAACGAGCGGCTCCTGAC TACTGAC	Eurofins Genomics; This paper	recC-KO-R1
GTCAGTAGTCAGGAGCCGCTCGTTTCCTGTTAC CGCTGTT	Eurofins Genomics; This paper	recC-KO-F2
AAAAGTCGACATTTAACTGCCTGCGGATCA	Eurofins Genomics; This paper	recC-KO-R2
AAAAACTGCAGGTTAATCCGCCAGTTTGACC	Eurofins Genomics; This paper	recD-KO-F1
CAATTACGTTTATTTCATTACGCCTCCTCCAG	Eurofins Genomics; This paper	recD-KO-R1
GGCGTAATGAAATAAACGTAATTGCCGGATGC	Eurofins Genomics; This paper	recD-KO-F2
AAAAAGTCGACGGAGCAGCAAGGTATTCTGG	Eurofins Genomics; This paper	recD-KO-R2
AAAAAGTCGACGCTGGTGGCACTGGGTAGT	Eurofins Genomics; This paper	attB::PsfiA-gfp F
AAAAAGCGGCCGCCAGGCATCACGGCAATATAC	Eurofins Genomics; This paper	attB::PsfiA-gfp R
GACCATGGTCTCCAATTCTTGGGATAACAGGGTA AAG	Eurofins Genomics; This paper	I-SceIcsSDM4F

CTTTACCCTGTTATCCCAAGAATTGGAGACCATG GTC	Eurofins Genomics; This paper	I-SceIcsSDM4R
AAAAACTGCAGATCGTCGTATCCCACTACCG	Eurofins Genomics; This paper	2357
AATAAGTATGTTGTAACTAAAGTGACCAGACACC CATCAACAG	Eurofins Genomics; This paper	2361
ACTTTAGTTACAACATACTTATTACATCAAGAAAT AACGCCGG	Eurofins Genomics; This paper	2362
AAAAAGTCGACCGATGGCGGAGCTGAATTAC	Eurofins Genomics; This paper	2360
AAAAACTGCAGGCATCAACAAACGGCTAAGG	Eurofins Genomics; This paper	YkgC-F1
AATGCGGTGCGGCCGCAAATCTGTGTGCTGCTG TGC	Eurofins Genomics; This paper	YkgC-R1
ACAGATTTGCGGCCGCACCGCATTGTACGTGAT GAG	Eurofins Genomics; This paper	YkgC-F2
AAAAAGTCGACTGTTCTGGCGTCTGATTTTG	Eurofins Genomics; This paper	YkgC-R2
AAAAAGTCGACGGTGATGTCGGCGATATAGG	Eurofins Genomics; This paper	pGB2F
ACGGCGTTTCACTTCTGAGT	Eurofins Genomics; This paper	pGB2R
GCGAACAGGGGCACAACTATAATCGTGAAGTGC TGG	Eurofins Genomics; This paper	Minus10_F
CCAGCACTTCACGATTATAGTTGTGCCCCTGTTC GC	Eurofins Genomics; This paper	Minus10_R
GGAAGCGCAGGGGATTGACAGCGAACAGGGGC AC	Eurofins Genomics; This paper	Minus35_F
GTGCCCCTGTTCGCTGTCAATCCCCTGCGCTTC C	Eurofins Genomics; This paper	Minus35_R
AAAAAGCGGCCGCGCAGCGCGTATTATCGAAC	Eurofins Genomics; This paper	FP_NotI_F

AAAAAGCGGCCGCAAGCTTTTATCTAGACTTGTACAGC	Eurofins Genomics; This paper	FP_NotI_R2
AAAAACTCGAGCCCGTCTGGTCTACATTTGG	Eurofins Genomics; This paper	hupA-mCherry_F2
AAAAAGCGGCCGCTGGTCGTTAGAAAGCTGCTG	Eurofins Genomics; This paper	hupA-mCherry_R2
AAAAAGTCGACTGCCCTTTAGAAGGGGAAAG	Eurofins Genomics; This paper	pMW11-R
TCTAGCAGGAGGAATTCACCA	Eurofins Genomics; This paper	TetR-mCherryA_F1
CGAGGATGTCAGACCCACTT	Eurofins Genomics; This paper	TetR-mCherryA_F1
GGTCTGACATCCTCGAGTTGGTTAGCAAAGGGGAAGAGTT	Eurofins Genomics; This paper	tetRYpet_coliF2
AAAAATCTAGATTACTTGTACAGCTCATTCATGC	Eurofins Genomics; This paper	tetRYpet_coliR2
GCGTGACTGGGATGAACC	(4)	DIGmalZ-F
CGGATCGTTTTTGCCATC	(4)	DIGmalZ-R
ATAGCGCTGGCTGCATCT	(4)	DIGperR-F
GCTGGGGGAAAACCAAAT	(4)	DIGperR-R
TAGCGGCTGATGTTGAACTG	(13)	lacZp.F
ATGAACGGTCTGGTCTTTGC	(13)	lacZp.R
TTACCAAACAGCCCGACTTC	(12)	ykgM-probeR
GGCCACAGCTAAAGCTAACC	(12)	3YkgM-F
**Antibodies, Recombinant Proteins and Commercial Kits**		
Anti-RecA	Abcam	Ab63797
Protein G Dynabeads®	ThermoFisher	Cat# 10003D
cOmplete™, mini, EDTA-free Protease Inhibitor Cocktail	Roche	Cat# 4693159001
PstI-HF	New England Biolabs	Cat# R3140S
SalI-HF	New England Biolabs	Cat# R3138S
NotI-HF	New England Biolabs	Cat# R3189S
XbaI	New England Biolabs	Cat# R0145S
NdeI	New England Biolabs	Cat# R0111S

PvuII-HF	New England Biolabs	Cat# R3151S
Quick Ligation Kit	New England Biolabs	Cat# M2200S
I-SceI	New England Biolabs	Cat# R0694S
RiboShredder RNase Blend	Epicentre	Cat# RS12100
Wizard Genomic DNA Purification Kit	Promega	Cat# A1120
MinElute PCR purification kit	Qiagen	Cat# 28004
NEBNext® ChIP-Seq Library Prep Master Mix Set for Illumina	New England Biolabs	Cat# E6240S
NEBNext® Multiplex Oligos for Illumina (Index Primers Sets 1 and 2)	New England Biolabs	Cat# E7335; Cat# E7500
Illumina TruSeq DNA Sample Prep Kit	Illumina	FC-121-3003
Prime-It II Random Labeling Kit	Agilent	Cat# 300385
**Software and Algorithms**		
MATLAB	Mathworks	vR2017a
R	N/A	https://www.r-project.org/
FIJI	(8)	https://fiji.sc/
ImageQuant	GE Healthcare	TL 7.01
MetaMorph	Molecular Devices	v7
Autoquant	Mediacy	X2
Burrows-Wheeler Aligner (BWA-MEM)	N/A	http://bio-bwa.sourceforge.net/
Bowtie 2	(10)	http://bowtie-bio.sourceforge.net/bowtie2/index.shtml
SAMtools	(9)	http://samtools.sourceforge.net/

Supplemental References

1. Khlebnikov, A., Datsenko, K.A., Skaug, T., Wanner, B.L. and Keasling, J.D. (2001) Homogeneous expression of the P(BAD) promoter in Escherichia coli by constitutive expression of the low-affinity high-capacity AraE transporter. *Microbiology*, **147**, 3241-3247.
2. White, M.A., Eykelenboom, J.K., Lopez-Vernaza, M.A., Wilson, E. and Leach, D.R. (2008) Non-random segregation of sister chromosomes in Escherichia coli. *Nature*, **455**, 1248-1250.
3. Merlin, C., McAteer, S. and Masters, M. (2002) Tools for characterization of Escherichia coli genes of unknown function. *J Bacteriol*, **184**, 4573-4581.

4. Eykelenboom, J.K., Blackwood, J.K., Okely, E. and Leach, D.R. (2008) SbcCD causes a double-strand break at a DNA palindrome in the Escherichia coli chromosome. *Mol Cell*, **29**, 644-651.
5. McCool, J.D., Long, E., Petrosino, J.F., Sandler, H.A., Rosenberg, S.M. and Sandler, S.J. (2004) Measurement of SOS expression in individual Escherichia coli K-12 cells using fluorescence microscopy. *Mol Microbiol*, **53**, 1343-1357.
6. Marceau, A.H., Bahng, S., Massoni, S.C., George, N.P., Sandler, S.J., Marians, K.J. and Keck, J.L. (2011) Structure of the SSB-DNA polymerase III interface and its role in DNA replication. *Embo J*, **30**, 4236-4247.
7. Churchward, G., Belin, D. and Nagamine, Y. (1984) A pSC101-derived plasmid which shows no sequence homology to other commonly used cloning vectors. *Gene*, **31**, 165-171.
8. Schindelin, J., Arganda-Carreras, I., Frise, E., Kaynig, V., Longair, M., Pietzsch, T., Preibisch, S., Rueden, C., Saalfeld, S., Schmid, B. *et al.* (2012) Fiji: an open-source platform for biological-image analysis. *Nat Methods*, **9**, 676-682.
9. Li, H., Handsaker, B., Wysoker, A., Fennell, T., Ruan, J., Homer, N., Marth, G., Abecasis, G., Durbin, R. and Genome Project Data Processing, S. (2009) The Sequence Alignment/Map format and SAMtools. *Bioinformatics*, **25**, 2078-2079.
10. Langmead, B. and Salzberg, S.L. (2012) Fast gapped-read alignment with Bowtie 2. *Nat Methods*, **9**, 357-359.
11. Anders, S. and Huber, W. (2010) Differential expression analysis for sequence count data. *Genome Biol*, **11**, R106.
12. Azeroglu, B., Mawer, J.S., Cockram, C.A., White, M.A., Hasan, A.M., Filatenkova, M. and Leach, D.R. (2016) RecG Directs DNA Synthesis during Double-Strand Break Repair. *PLoS Genet*, **12**, e1005799.
13. Mawer, J.S. and Leach, D.R. (2014) Branch migration prevents DNA loss during double-strand break repair. *PLoS Genet*, **10**, e1004485.
14. Azeroglu, B., Lincker, F., White, M.A., Jain, D. and Leach, D.R. (2014) A perfect palindrome in the Escherichia coli chromosome forms DNA hairpins on both leading- and lagging-strands. *Nucleic Acids Res*, **42**, 13206-13213.
15. Cockram, C.A., Filatenkova, M., Danos, V., El Karoui, M. and Leach, D.R. (2015) Quantitative genomic analysis of RecA protein binding during DNA double-strand break repair reveals RecBCD action in vivo. *Proc Natl Acad Sci U S A*, **112**, E4735-4742.
